# Plasma proteomic signature of decline in gait speed and grip strength

**DOI:** 10.1111/acel.13736

**Published:** 2022-11-04

**Authors:** Xiaojuan Liu, Stephanie Pan, Vanessa Xanthakis, Ramachandran S. Vasan, Bruce M. Psaty, Thomas R. Austin, Anne B. Newman, Jason L. Sanders, Chenkai Wu, Russell P. Tracy, Robert E. Gerszten, Michelle C. Odden

**Affiliations:** ^1^ Department of Epidemiology and Population Health Stanford University School of Medicine Stanford California USA; ^2^ Framingham Heart Study and Section of Preventive Medicine and Epidemiology Boston University School of Medicine Boston Massachusetts USA; ^3^ Department of Biostatistics Boston University School of Public Health Boston Massachusetts USA; ^4^ Department of Epidemiology Boston University School of Public Health Boston Massachusetts USA; ^5^ Section of Cardiovascular Medicine, Department of Medicine Boston University School of Medicine Boston Massachusetts USA; ^6^ Cardiovascular Health Research Unit, Departments of Medicine, Epidemiology, and Health Systems and Population Health University of Washington Seattle Washington USA; ^7^ Department of Epidemiology University of Washington Seattle Washington USA; ^8^ Department of Epidemiology University of Pittsburgh Pittsburgh Pennsylvania USA; ^9^ Vertex Pharmaceuticals Inc Boston Massachusetts USA; ^10^ Global Health Research Center Duke Kunshan University Kunshan China; ^11^ Department of Pathology and Laboratory Medicine, The Robert Larner M.D. College of Medicine University of Vermont Burlington Vermont USA; ^12^ Department of Biochemistry, The Robert Larner M.D. College of Medicine University of Vermont Burlington Vermont USA; ^13^ Division of Cardiovascular Medicine Beth Israel Deaconess Medical Center Boston Massachusetts USA

**Keywords:** aging, gait speed, grip strength, physical function, proteomics

## Abstract

The biological mechanisms underlying decline in physical function with age remain unclear. We examined the plasma proteomic profile associated with longitudinal changes in physical function measured by gait speed and grip strength in community‐dwelling adults. We applied an aptamer‐based platform to assay 1154 plasma proteins on 2854 participants (60% women, aged 76 years) in the Cardiovascular Health Study (CHS) in 1992–1993 and 1130 participants (55% women, aged 54 years) in the Framingham Offspring Study (FOS) in 1991–1995. Gait speed and grip strength were measured annually for 7 years in CHS and at cycles 7 (1998–2001) and 8 (2005–2008) in FOS. The associations of individual protein levels (log‐transformed and standardized) with longitudinal changes in gait speed and grip strength in two populations were examined separately by linear mixed‐effects models. Meta‐analyses were implemented using random‐effects models and corrected for multiple testing. We found that plasma levels of 14 and 18 proteins were associated with changes in gait speed and grip strength, respectively (corrected *p* < 0.05). The proteins most strongly associated with gait speed decline were GDF‐15 (Meta‐analytic *p* = 1.58 × 10^−15^), pleiotrophin (1.23 × 10^−9^)*,* and TIMP‐1 (5.97 × 10^−8^). For grip strength decline, the strongest associations were for carbonic anhydrase III (1.09 × 10^−7^), CDON (2.38 × 10^−7^), and SMOC1 (7.47 × 10^−7^). Several statistically significant proteins are involved in the inflammatory responses or antagonism of activin by follistatin pathway. These novel proteomic biomarkers and pathways should be further explored as future mechanisms and targets for age‐related functional decline.

## Graphical abstract

Several plasma proteins were associated with physical function decline in adults from middle to older age, including GDF‐15, pleiotrophin, and TIMP‐1 for gait speed decline, and CA III, CDON, and SMOC1 for grip strength decline. These novel proteomic biomarkers may by explored as future mechanisms and targets for age‐related functional decline.

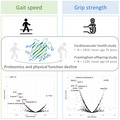

**Abbreviations**
3MSE: modified mini‐mental state examination
ANML: adaptive normalization by maximum likelihood
BMI: body mass index
CHS: cardiovascular Health Study
COPD: chronic obstructive pulmonary disease
CVD: Cardiovascular disease
eGFR: estimating glomerular filtration rate
FOS: framingham offspring study
LMER: linear mixed‐effects regressions
SOMA: slow off‐rate modified aptamers

## 1 INTRODUCTION

Gait speed and grip strength are well‐established physical function measures predictive of health‐related quality of life among older adults (Fried et al., 2001; Perera et al., 2016). Decline in gait speed and grip strength may contribute to frailty, disability, morbidity, and mortality and serve as a marker of progressive loss of independence (Vermeulen et al., 2011; White et al., 2013). Prevention of physical decline with aging is important to maintain functional independence and peak quality of life across the life course.

Optimal prevention of functional decline may be aided by a more complete understanding of its antecedent mechanisms. To date, incident functional decline has been associated with a number of environmental, social, and behavioral risk factors (Plouvier et al., 2016; Stenholm et al., 2012; Sternäng et al., 2015) as well as pathologies in multiple tissues and organs (Kim et al., 2015; Rosano et al., 2005). Yet, the biologic mechanisms mediating these relationships remain incompletely described. Moreover, function declines even in apparently healthy older adults (Elstgeest et al., 2020), highlighting the significance of identifying early biomarkers in the predisability state. Several candidate biomarkers, such as interleukin‐6, dehydroepiandrosterone sulfate, and insulin‐like growth hormone, have been associated with age‐related changes in physical function (Newman et al., 2016; Sanders et al., 2014). However, prior studies were based on data which included relatively few biomarkers or selected pathways, typically associated these with single measurements of physical function, and lacked replication in external cohorts. More reliable or actionable conclusions might be generated from data, which includes a broader set of biomarkers, characterizes longitudinal trajectories of physical function, and includes replication.

Proteins are direct effectors of many biological mechanisms and often final determinants of phenotypes that provide insights into complex biological processes. Subsequently, proteins may be particularly valuable biomarkers in the searching for causal or predictive associations with important human phenotypes. Recent advances in aptamer‐based platforms allow investigators to measure thousands of proteins simultaneously in small samples and have facilitated proteomic research to discover biological and pathophysiologic pathways involved in human disease. Indeed, proteomic approaches have been applied to identify new frailty or disability biomarkers (Darvin et al., 2014; Osawa et al., 2020; Sathyan, Ayers, Gao, Milman, et al., 2020; Sathyan, Ayers, Gao, Weiss, et al., 2020; Shamsi et al., 2012). Despite these advances, no large‐scale proteomics studies have evaluated age‐related decline in physical function as measured by gait speed and grip strength. A better understanding of the proteomic profile of physical function may inform targets for interventions to effectively prevent and slow the clinical course of physical decline and provide insights into the biology of frailty, disability, and aging on a population level.

The Cardiovascular Health Study (CHS) and Framingham Offspring Study (FOS) are two population‐based cohorts with measurements of longitudinal physical functions in a large sample of adults with a broad age range. In this investigation, we leveraged these data and conducted the proteomic analysis using the SOMAscan (Gold et al., 2010) assay that measured over 1100 proteins in 3984 adults from these two large longitudinal cohorts. The goal was to characterize the plasma proteomic profile of long‐term changes in gait speed and grip strength in community‐dwelling adults in midlife and older age. We aimed to better capture the biology underlying decline in physical function and identify novel proteomic biomarkers for physical decline.

## 2 METHODS

### 2.1 Study populations

#### 2.1.1 Cardiovascular Health Study

The CHS is a prospective cohort study of community‐dwelling individuals 65 years of age or older from four US communities: Sacramento County, CA; Washington County, MD; Forsyth County, NC; and Allegheny County, PA. The study recruited an initial cohort of 5201 men and women in 1989–1990, and an additional 687 African Americans were recruited in 1992–1993. Participants underwent extensive annual clinical examinations, which measured traditional cardiovascular risk factor and measures of subclinical disease, as well as phone interviews at 6‐month intervals through 1999. Each participant provided written informed consent, and Institutional Review Boards (IRB) approved the study protocol at each site (Fried et al., 1991).

Plasma proteins were measured in 3185 participants who had available unthawed samples in 1992–1993. For this analysis, participants were followed for 6 years from 1992–1993 (baseline) through 1998–1999. We excluded participants who had no proteomics data or had no measurements for gait speed or grip strength, or had missingness on covariates, resulting in an analytic sample of 2854 participants (Figure S1).

#### 2.1.2 Framingham Offspring Study

The FOS cohort was established in 1971, consisting of the children of the original Framingham Heart Study (FHS) cohort and their spouses (Feinleib et al., 1975). The FOS cohort completes a series of questionnaires and laboratory and cardiovascular tests and undergoes a physical examination every 4 years. The details of the sampling methods and design of the study have been previously published (Kannel et al., 1979). All study participants provided informed consent, and protocols were approved by the IRB at Boston University Medical Center (Boston, MA).

The present investigation utilized data derived from the FOS cohort fifth (1991–1995, baseline), seventh (1998–2001), and eighth (2005–2008) examination cycles. There were 1913 participants who had completed proteomic profiling at the fifth exam and were eligible for this investigation. The same exclusion criteria were applied to FOS cohort, resulting in an analytic sample of 1130 participants (Figure S2). No major differences were detected between those who were included and who were excluded for both cohorts (Table S1).

### 2.2 Covariates

Sociodemographic factors, including age, sex, Black race (for CHS only), years of education, and smoking status (current/former/never), were determined by self‐report in both cohorts. Weight (kg) and height (cm) were assessed using a standard protocol, and body mass index (BMI) was calculated as kg/m^2^. Blood pressure was recorded as the average of two measurements. Estimating glomerular filtration rate (eGFR) was calculated by CKD‐EPI 2012 equation based on cystatin C measurements. Medication use was determined by a medication/drug inventory. Diabetes mellitus was defined as the use of insulin or oral hypoglycemic medications or fasting serum glucose ≥ 126 mg/dl or nonfasting serum glucose ≥200 mg/dl. Modified Mini‐Mental State Examination (3MSE) scores were used as an evaluation of cognitive function in CHS. Cardiovascular disease (CVD, including coronary heart disease, heart failure, peripheral arterial disease [for FOS], and stroke) were identified by participant report or proxy report, confirmed by medical record review and adjudicated according to standard criteria. Atrial fibrillation, chronic obstructive pulmonary disease (COPD), cancer, and arthritis were identified by participant or proxy report and/or medical record. Clinic site (Bowman Gray, Johns Hopkins, Davis, and Pittsburgh) was also included as a covariate in CHS. The covariates selected for inclusion in our analysis were identified as critical confounders in associations with physical function (Plouvier et al., 2016; Stenholm et al., 2012; Sternäng et al., 2015) and included in previous studies of proteins with aging outcomes (Osawa et al., 2020; Sathyan, Ayers, Gao, Milman, et al., 2020; Sathyan, Ayers, Gao, Weiss, et al., 2020).

### 2.3 Proteomics

The SOMAscan assays (SOMALogic) have been described previously (Mehan et al., 2013). Briefly, slow off‐rate modified aptamers (SOMA) were used to target proteins and evaluated relative concentrations of multiple proteins on a single assay and SOMAscan was preferred due to a larger panel size over other platforms at the time of the study. The clinical and analytic validity of the aptamer‐based assay method has been demonstrated in recent reports (Ngo et al., 2016) and the high correlation between SOMAscan levels and conventional ELISA‐based measures suggests the platform is comparable to other available methods and particularly well suited for plasma biomarker discovery (Austin et al., 2022; Fitzgibbons et al., 2017). The SOMAscan technology was used to run the assays in 2020 for CHS (Version 4.0) and in 2011 for FOS (Version 1.1 and 1.3) and, according to the fee for service agreement, provided measures of 1305 plasma proteins in CHS from samples drawn in 1992–1993 (the third examination for the original cohort and first for the African American cohort) and 1373 proteins in FOS from samples drawn in 1991–1995 (the fifth examination). A total of 1154 proteins were measured in both cohorts and included in the present study (a complete list of the proteins is included in Table S2). Adaptive normalization by maximum likelihood (ANML) and median normalization by plate were performed in CHS and FOS, respectively, in order to remove sample or assay biases. The median intra‐assay (inter‐assay) coefficient of variation was 3.4% (4.4%) for CHS proteins using quality control samples and <8.2% (7.8%) for FOS proteins using pooled plasma samples. Intraclass correlation coefficient was 0.66 in 100 samples from two examination cycles for CHS and >0.95 for FOS‐blinded duplicate samples (Austin et al., 2022; Ngo et al., 2016). The values of protein levels were log_2_‐transformed and standardized (mean = 0, *SD* = 1) in CHS and were log_e_‐transformed and standardized in FOS for this analysis.

### 2.4 Physical function outcomes

In CHS, physical function measures were assessed annually for 7 years. Gait speed (meters per second) was measured over 15‐foot (4.6 m) course at normal pace. Grip strength (kilograms) was measured using an adjustable Jamar isometric handheld dynamometer (JAMAR Technologies, Inc.) and calculated as an average of three readings in the dominant hand.

In FOS, physical function was measured twice in seventh (1998–2001) and eighth (2005–2008) examination cycles, approximately 7 years apart. Gait speed (meters per second) was measured over a 4‐m course at usual pace. The test was completed twice with the faster record used for analysis. Grip strength (kilograms) was measured using a Jamar handheld dynamometer. Three trials were attempted on each hand for a total of up to six trials, and the maximum value among all trials, regardless of side, was used in the analyses.

### 2.5 Statistical analysis

Linear mixed‐effects regressions (LMER) were used to examine the associations of individual protein levels with longitudinal changes in gait speed and grip strength separately in CHS and FOS samples. LMER were performed with repeated measurements of gait speed or grip strength as outcomes, participant identification as random intercepts, and follow‐up time (years), individual protein level, and time × protein interaction as fixed effects. The models were primarily adjusted for age, sex, education, height, weight, BMI, smoking status, and eGFR (CHS models also included race and clinic site) to control for potential confounding. Blood pressure, antihypertensive medication, cognitive function, and chronic conditions (e.g., diabetes, CVD, arthritis, and atrial fibrillation) were further adjusted as secondary confounders or potential mediators. Interactions of the sex term with time were also assessed to allow for different rates of physical decline over time by sex. The results from the LMER were expressed as the beta coefficients of the protein × time interaction terms, associated with 1 standard error increment of the log‐transformed protein biomarker per year. Since the physical function declined over time (coefficient of the time term was negative), a positive and a negative coefficient of the time × protein term should be interpreted as an association with a slower and faster decline in physical function, respectively. In sensitivity analyses, we excluded those with prevalent stroke for both samples (*n* = 104 in CHS and *n* = 1 in FOS) considering that stroke is known as a highly disabling event and often causes difficulties in ambulation. We also evaluated a model additionally adjusted for a baseline age × time interaction term to uncover the potential effect modification of age on physical decline trajectories.

Meta‐analyses were implemented using random‐effects models in the R package metafor to combine two effect sizes from both samples and characterize a composite coefficient for each protein. No major heterogeneity was detected between effect sizes of two samples (low *τ* ^2^ and *I* ^2^). Multiple testing was corrected by the Bonferroni method using the number of principal components explaining 95% of the total variability in proteins, corresponding to a significance threshold of 0.05/742 = 6.7 × 10^−5^. Volcano plots were adopted to display the effect size of each individual protein and negative log_10_‐transformed *p*‐values. Statistical analyses were conducted in R, version 3.2.4 and SAS version 9.4 (Cary, NC). Pathway analysis was carried out using Reactome (www.reactome.org), a curated database of pathways and reactions in human biology, for the proteins that reached the statistically significant threshold in the primary analysis. This database was queried with the UniProt IDs to perform the statistical (hypergeometric distribution) test that determines whether certain Reactome pathways are over‐represented (Fabregat et al., 2018).

## 3 RESULTS

A total of 2854 CHS participants (60% women; mean age, 76.3 ± 5.0 years) and 1130 FOS participants (55% women; mean age, 54.2 ± 9.5 years) were included in the primary analysis, ranging in age from 29 to 100 years (Figure S3). Demographic and clinical baseline characteristics of the two samples are summarized in Table 1. Overall, FOS participants had greater BMI and eGFR and were more likely to be current or former smokers and less likely to have chronic conditions. Table S3 presents the unadjusted physical function declines during follow‐up in two samples. Participants in CHS had lower baseline levels and faster declines in gait speed and grip strength than participants in FOS, reflecting the overall older age of CHS participants compared with FOS participants.

**TABLE 1:** Baseline characteristics in CHS and FOS.

Variables	CHS (*n* = 2854)	FOS (*n* = 1130)
Study year	1992–3	1991–5
Age, years	76.3 (5.0)	54.2 (9.5)
Sex, female	1717 (60.2%)	617 (54.6%)
Race		
Black	419 (14.7%)	0
White/other	2435 (85.3%)	1130 (100.0%)
Clinic		
Bowman gray	734 (25.7%)	
Davis	717 (25.1%)	
Hopkins	627 (22.0%)	
Pittsburgh	776 (27.2%)	
Education, years	12.6 (2.8)	14.2 (2.5)
Height, cm	164.3 (9.5)	167.5 (9.4)
Weight, kg	71.9 (14.1)	77.1 (16.1)
BMI, kg/m^2^	26.6 (4.5)	27.4 (4.9)
Smoking status		
Current	264 (9.3%)	199 (17.6%)
Former	1271 (44.5%)	576 (51.0%)
Never	1319 (46.2%)	355 (31.4%)
SBP, mmHg	135.4 (21.2)	125.2 (18.6)
DBP, mmHg	71.1 (10.9)	74.3 (10.1)
Gait speed, m/s	0.9 (0.2)	1.2 (0.3)^a^
Grip strength, kg	28 (9.8)	32.8 (13.0)^a^
eGFR, ml/min/1.73 m^2^	67.2 (17.4)	90.0 (19.4)
3MSE. points	91.6 (7.7)	–
Diabetes status		
Normal	2190 (76.7%)	1068 (94.5%)
Diabetes	385 (13.5%)	62 (5.5%)
Impaired fasting glycemia	279 (9.8%)	–
CVD	598 (21.0%)	55 (4.9%)
Atrial fibrillation	141 (4.9%)	11 (1.0%)
COPD	330 (11.6%)	–
Cancer	117 (4.1%)	191 (16.9%)
Arthritis	1266 (44.4%)	–
Hypertension medication	1342 (47.0%)	187 (16.5%)

*Note*: Data were mean (standard deviation) or *n* (%).

Abbreviation: 3MSE, Modified Mini‐Mental State Examination; CHS, Cardiovascular Health Study; COPD, chronic obstructive pulmonary disease; CVD, cardiovascular disease; DBP, diastolic blood pressure; eGFR, estimated glomerular filtration rate; FOS, Framingham Offspring Study; SBP, systolic blood pressure.

^a^ Reported measures are obtained from the seventh examination cycle in FOS.

Table 2 shows the 14 proteins that were significantly associated with gait speed decline by meta‐analysis. Of these, 13 have negative coefficients, indicating that higher concentrations of these proteins associated with greater gait speed decline, and one has a positive coefficient, indicating higher protein level was associated with slower gait speed decline. The three strongest associations between protein level and greater gait speed decline were for growth/differentiation factor 15 (GDF‐15, alias MIC‐1; Meta‐analytic *β* [*SE*] = −0.0055 [0.0007]; unadjusted *p* = 1.58 × 10^−15^), pleiotrophin (PTN) (β [*SE*] = −0.0041 [0.0007]; *p* = 1.23 × 10^−9^), and metalloproteinase inhibitor 1 (TIMP‐1) (*β* [*SE*] = −0.0035 [0.0006]; *p* = 5.97 × 10^−8^). The protein associated with a slower gait speed decline was interleukin‐7 receptor subunit alpha (IL‐7a) (*β* [*SE*] = 0.0035 [0.0007]; *p* = 7.97 × 10^−8^).

**TABLE 2:** Significant SOMAscan proteins associated with gait speed decline by meta‐analysis.

SeqId	SomaId	UniProt	Target	Target full name	Estimates	*SE*	*p*‐value
4374‐45	SL003869	Q99988	GDF‐15	Growth/differentiation factor 15	−0.0055	0.0007	1.58 E‐15
3045‐72	SL002704	P21246	PTN	Pleiotrophin	−0.0041	0.0007	1.23 E‐09
2211‐9	SL000591	P01033	TIMP‐1	Metalloproteinase inhibitor 1	−0.0035	0.0006	5.97 E‐08
5315‐22	SL000052	P45379	Troponin T	Troponin T, cardiac muscle	−0.0038	0.0007	7.04 E‐08
5089‐11	SL005189	P16871	IL‐7 Ra	Interleukin‐7 receptor subunit alpha	0.0035	0.0007	7.97 E‐08
6649‐51	SL012395	O95631	NET1	Netrin‐1	−0.0033	0.0006	1.32 E‐07
3044‐3	SL003323	P55774	PARC	C‐C motif chemokine 18	−0.0029	0.0006	2.25 E‐06
2944‐66	SL005156	P41271	DAN	Neuroblastoma suppressor of tumorigenicity 1	−0.0033	0.0007	4.38 E‐06
2602‐2	SL001996	O15123	Angiopoietin‐2	Angiopoietin‐2	−0.0027	0.0006	1.76 E‐05
3799‐11	SL004867	P07451	Carbonic anhydrase III	Carbonic anhydrase 3	−0.0027	0.0006	2.21 E‐05
3438‐10	SL009324	O95633	FSTL3	Follistatin‐related protein 3	−0.0027	0.0007	3.70 E‐05
3234‐23	SL010390	Q76M96	URB	Coiled‐coil domain‐containing protein 80	−0.0026	0.0006	4.74 E‐05
11514‐196	SL004557	P13987	CD59	CD59 glycoprotein	−0.0028	0.0007	5.18 E‐05
13094‐75	SL018509	Q9BXY4	RSPO3	R‐spondin‐3	−0.0026	0.0007	5.87 E‐05

*Note*: Estimates were from linear mixed effect models adjusted for age, sex, sex × time, race, clinic, education, height, weight, BMI, smoking status, and eGFR. Significant threshold was 6.7 × 10^−5^.

**FIGURE 1:**
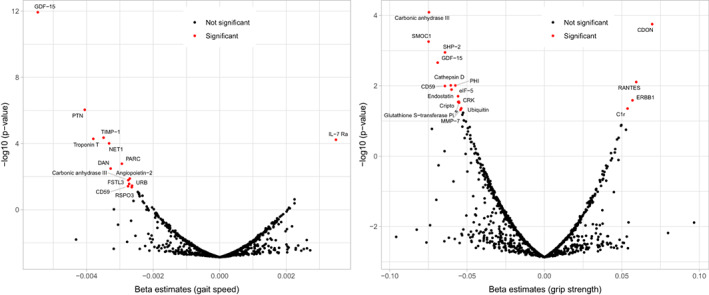
Volcano plot summarizing associations of 1154 plasma proteins with longitudinal changes in gait speed (left) and grip strength (right). Beta coefficients and *p*‐values are from linear mixed effect models adjusted for age, sex, sex × time, race, clinic, education, height, weight, BMI, smoking status, and eGFR. Significant proteins (*p*‐value < 6.7 × 10^−5^) are annotated and shown as red dots.

Table 3 shows the 18 proteins that were significantly associated with grip strength decline by meta‐analysis. Of these, 14 have negative coefficients and four have positive coefficients. The top three protein‐grip change associations were for carbonic anhydrase III (CA3) (*β* [*SE*] = −0.0746 [0.0141]; *p* = 1.09 × 10^−7^), cell adhesion molecule‐related/downregulated by oncogenes (CDON) (*β* [*SE*] = 0.0696 [0.0135]; *p* = 2.38 × 10^−7^), and SPARC‐related modular calcium‐binding protein 1 (SMOC1) (*β* [*SE*] = −0.0749 [0.0151]; *p* = 7.47 × 10^−7^), wherein higher level of CDON was associated with slower grip strength decline. The other three proteins associated with slower grip strength decline were C‐C motif chemokine 5 (RANTES), epidermal growth factor receptor (ERBB1), and complement C1r subcomponent (C1r). It is notable that GDF‐15 was strongly associated with not only gait speed decline but also grip decline (*β* [*SE*] = −0.0690 [0.0148]; *p* = 2.96 × 10^−6^), and CA3 and CD59 glycoprotein (CD59) were also statistically significantly associated with both outcomes. The volcano plots displaying the associations of 1154 plasma proteins with decline in gait speed and grip strength are shown in Figure 1.

**TABLE 3:** Significant SOMAscan proteins associated with grip strength decline by meta‐analysis.

SeqId	SomaId	UniProt	Target	Target full name	Estimates	*SE*	*p*‐value
3799‐11	SL004867	P07451	Carbonic anhydrase III	Carbonic anhydrase 3	−0.0746	0.0141	1.09 E‐07
4541‐49	SL014092	Q4KMG0	CDON	Cell adhesion molecule‐related/downregulated by oncogenes	0.0696	0.0135	2.38 E‐07
13118‐5	SL011888	Q9H4F8	SMOC1	SPARC‐related modular calcium‐binding protein 1	−0.0749	0.0151	7.47 E‐07
3397‐7	SL010616	Q06124	SHP‐2	Tyrosine‐protein phosphatase nonreceptor type 11	−0.0642	0.0134	1.51 E‐06
4374‐45	SL003869	Q99988	GDF‐15	Growth/differentiation factor 15	−0.0690	0.0148	2.96 E‐06
5480‐49	SL000563	P13501	RANTES	C‐C motif chemokine 5	0.0593	0.0135	1.05 E‐05
5508‐62	SL000344	P07339	Cathepsin D	Cathepsin D	−0.0605	0.0139	1.31 E‐05
4272‐46	SL000539	P06744	PHI	Glucose‐6‐phosphate isomerase	−0.0576	0.0132	1.32 E‐05
11514‐196	SL004557	P13987	CD59	CD59 glycoprotein	−0.0643	0.0148	1.36 E‐05
2201‐17	SL000403	P39060	Endostatin	Endostatin	−0.0600	0.0140	1.72 E‐05
2612‐5	SL004759	P55010	eIF‐5	Eukaryotic translation initiation factor 5	−0.0558	0.0133	2.66 E‐05
2677‐1	SL002644	P00533	ERBB1	Epidermal growth factor receptor	0.0569	0.0137	3.48 E‐05
4976‐57	SL013240	P46108	CRK	Adapter molecule crk	−0.0553	0.0134	3.81 E‐05
5810‐25	SL005155	P13385	Cripto	Teratocarcinoma‐derived growth factor 1	−0.0557	0.0136	3.89 E‐05
4911‐49	SL003643	P09211	Glutathione S‐transferase Pi	Glutathione S‐transferase P	−0.0552	0.0134	4.06 E‐05
4474‐19	SL004070	P62979	Ubiquitin	Ubiquitin	−0.0537	0.0134	5.87 E‐05
3285‐23	SL000310	P00736	C1r	Complement C1r subcomponent	0.0537	0.0134	5.96 E‐05
2789‐26	SL000525	P09237	MMP‐7	Matrilysin	−0.0542	0.0136	6.48 E‐05

*Note*: Estimates are from linear mixed effect models adjusted for age, sex, sex × time, race, clinic, education, height, weight, BMI, smoking status, and eGFR. Significant threshold was 6.7 × 10^−5^.

After full adjustment for secondary confounders and potential mediators, the proteins significantly associated with gait speed and grip strength decline remained largely unchanged although the ordering by significance slightly shuffled, and secreted and transmembrane protein 1 (SECTM1) reached the statistical significance for grip model (Tables S4 and S5). When restricting to participants without prevalent stroke in a sensitivity analysis, the significant proteins remained similar for gait speed decline, although follistatin‐related protein 3 and CD59 no longer reached the threshold for significance (Table S6). For grip strength, several marginally significant proteins (ERBB1, adapter molecule crk, teratocarcinoma‐derived growth factor 1, complement C1r subcomponent, glutathione S‐transferase P) were no longer significant after excluding stroke participants and C‐X‐C motif chemokine 9 and Beta‐2‐microglobulin reached the statistical significance threshold (Table S7). When additionally adjusted for age × time interaction, PTN was no longer statistically significantly associated with change in gait speed, the other four of the original top five proteins remained significant and IL‐7 interleukin‐36 alpha additionally reached the significant threshold (Table S8). For grip strength decline, seven out of the original 18 significant proteins reached the threshold for statistical significance and CA3 remained the top (Table S9).

As a validation analysis, we looked into the GDF‐15 and TIMP‐1 measured with ELISA in FOS examination cycle 6 (1995–1998) and examined their associations with physical function declines. The results showed that ELISA‐measured GDF‐15 was significantly associated with greater gait speed decline (*β* [*SE*] = −0.0043 [0.0021], *p* = 0.0379) but not with grip decline (*β* [*SE*] = −0.0509 [0.0480], *p* = 0.2893). ELISA‐measured TIMP‐1 was not associated with either levels or changes in these two physical functions. We observed a correlation between SOMAscan measures with ELISA measures for GDF‐15 (sex and age‐adjusted Spearman *r* = 0.55, *p* < 0.001) but not for TIMP‐1 (*r* = 0.03, *p* = 0.39).

Reactome pathway analysis (including 14 significant proteins for gait speed and 18 for grip strength) found that “Antagonism of Activin by Follistatin” pathway was most strongly associated with gait speed decline, followed by pathways involved in post‐translational protein phosphorylation, RUNX1 regulates transcription of genes involved in WNT signaling and insulin‐like growth factor (IGF) transport and regulation. The pathway “Interleukin‐3, Interleukin‐5 and GM‐CSF signaling” that regulates varied inflammatory responses was found to be most strongly associated with grip strength decline in Reactome database, followed by pathways involved in signaling by EGFR remodeling, non‐receptor tyrosine kinases, PTK6, and collagen degradation (Table S7).

## 4 DISCUSSION

The present study identified proteomic profiles associated with change in physical function measures in adults in midlife and older age from two longitudinal cohort studies. Among 1154 plasma proteins quantified, 14 and 18 proteins were significantly associated with gait speed and grip strength decline in CHS and FOS, and GDF‐15, CA3 (Carbonic anhydrase III), and CD 59 (CD59 glycoprotein) are three proteins associated with both outcomes. To our knowledge, this is the first study to elucidate the proteomic signature of declines in gait speed and grip strength from middle to older age. The results of an untargeted proteomic approach may offer new insights into the pathogenesis and biomarkers of physical decline.

Several proteomic studies using the SOMAscan platform have investigated the proteomic profiles of aging‐related outcomes and reported associated observation of GDF‐15, PTN, spondin‐1, URB, FSTL3, and SMOC1 with frailty and mobility disability, and mortality, in older people (Osawa et al., 2020; Sathyan, Ayers, Gao, Milman, et al., 2020; Sathyan, Ayers, Gao, Weiss, et al., 2020). Of these, GDF‐15, a stress‐induced cytokine and a divergent member of the transforming growth factor (TGF)‐β superfamily, was the protein most strongly associated with physical function decline in our study. Recent studies have consistently demonstrated the upregulation of GDF‐15 in aging (Tanaka et al., 2018) and showed that higher GDF‐15 relates to higher risk of diabetes, cancer, cognitive impairment, cardiovascular diseases, and mortality (Justice et al., 2018). It has been proposed that GDF‐15 is a stress‐induced cytokine in response to tissue injury and can be utilized as a biomarker for various diseases (Emmerson et al., 2018). An Italian cohort study showed that GDF‐15 is associated with the development of mobility disability in community‐dwelling older adults (Osawa et al., 2020). Furthermore, investigators from the Baltimore Longitudinal Study of Aging (BLSA) found that elevated plasma GDF‐15 was associated with slower gait speed and low physical performance but not with muscle strength in community‐dwelling adults (Semba et al., 2020). However, this study included very healthy adults and adopted a cross‐sectional design and therefore is limited in revealing biomarkers for longitudinal changes in physical function. More work is needed to further elucidate the underlying molecular mechanisms of GDF‐15 in functional decline.

Carbonic anhydrase III (CA3) was also strongly associated with both gait speed and grip strength declines in our analysis. CA3 is a member of a multigene family that encodes carbonic anhydrase isozymes. It is strictly tissue‐specific and the muscle‐specific CA3 presents a sensitive biomarker of muscle damage and muscle pathology (Brancaccio et al., 2010). This enzyme has not been previously identified by aptamer‐based proteomic surveys of human aging and frailty. Nevertheless, recent mass spectrometry‐based proteomic and independent immunoblot analysis has suggested that CA3 levels increase in senescent human skeletal muscle and decrease in nonobese, diabetic skeletal muscle (Mullen & Ohlendieck, 2010; Staunton et al., 2012). The present results further suggest the independent effects of the CA3 on the physical function decline.

Pleiotrophin (PTN) was ranked second, and metalloproteinase inhibitor 1 (TIMP‐1) was ranked third among proteins significantly associated with gait speed decline in our study. PTN, also called osteoblast‐specific factor 1, is a heparin‐binding angiogenic growth factor and is involved in cell growth and survival, cell migration, and angiogenesis. It was shown that PTN increased in serum of patients during fracture healing and in the synovial fluid of patients with osteoarthritis (Pufe et al., 2003). Moreover, PTN overexpression was shown to enhance bone formation and regeneration and promote osteoprogenitor differentiation and proliferation (Lamprou et al., 2014). Tissue inhibitors of metalloproteinases (TIMPs) are a family of naturally occurring, endogenous inhibitors that participate in the inhibition and activation matrix metalloproteinase. There is evidence that TIMPs can also participate in other nontraditional functions such as cell proliferation, apoptosis, and angiogenesis (Nagase et al., 2006). It plays an anti‐apoptotic function and involves in age‐associated renal sclerotic and impairment kidney angiogenesis (Tan & Liu, 2012). The functionality and mechanism of action of PTN and TIMP‐1 are not completely elucidated.

We also found that CDON (cell adhesion molecule‐related/downregulated by oncogenes), the component of a cell‐surface receptor complex that mediates muscle precursor cell–cell interactions, was associated with a slower grip strength decline. Additionally, SMOC1, a member of the SPARC family and an important regulator of osteoblast differentiation (Choi et al., 2010), was associated with a greater grip strength decline. In an investigation of age‐related proteome profiles (Lehallier et al., 2019), Lehallier et al. reported the significant increase in SMOC1 and decrease in CDON across lifespan, suggesting the critical roles they played in aging process. Other proteins associated with aging outcomes that were confirmed in our study included troponin T, a marker of myocardial injury, URB, a regulator of cell adhesion and matrix assembly and FSTL3, a regulator of activin type II receptor signaling which relates to aging and frailty (Johnson et al., 2020; Roh et al., 2019; Sathyan, Ayers, Gao, Milman, et al., 2020; Sathyan, Ayers, Gao, Weiss, et al., 2020). On the contrary, several antibody‐based protein measures that were found statistically significant in previous CHS studies on functional changes, such as interleukin‐6 (IL‐6) and cystatin C (Newman et al., 2016; Sanders et al., 2014), were not observed to be statistically significant in this study. The reasons could be the moderate‐to‐low correlation between those SOMAscan and ELISA measures and the adjustment for eGFR attenuated the associations of kidney‐related biomarkers. The meta‐analysis approach with a secondary cohort may also dilute these associations.

In addition, we identified several other proteins associated with functional decline that have not been previously described. For example, IL‐7 Ra (interleukin‐7 receptor subunit alpha), a receptor for interleukin‐7 as well as thymic stromal lymphopoietin, and PARC (C‐C motif chemokine 18), a chemotactic factor involved in inflammation, were associated with gait speed decline; SHP‐2 (Tyrosine‐protein phosphatase nonreceptor type 11), which regulates skeletal cell lineage differentiation via dephosphorylates SOX9 (Zuo et al., 2018), and RANTES (C‐C motif chemokine 5), which stimulates inositol trisphosphate production and calcium mobilization (Liu et al., 2013), were associated with grip strength decline in our study. Additionally, CD59 (CD59 glycoprotein), a potent inhibitor of the complement membrane attack complex, was associated with both gait and grip decline. Although regarded as age‐related outcomes, it is notable that declines in gait speed and grip strength are different phenotypes and may share distinct physiological mechanism with the aging process. Gait is a higher‐order function integrates information on multiple physiologic systems, including the central and peripheral nervous systems, perceptual systems, musculoskeletal system, and energy production and/or delivery (Ferrucci et al., 2000), whereas grip strength is primarily a measure used in defining dynapenia and not as well established as gait speed as a predictor of clinical outcomes (Stessman et al., 2017). These differences between aging outcomes may explain why some significant biomarkers, such as N‐terminal pro‐BNP, that have been previously reported in aging studies were not found to be associated with our outcomes in this study.

The strengths of our study include the longitudinal design in two well‐characterized, large, population‐based cohorts. More importantly, this is the first study to date examining the associations of the protein panel targeted using the SOMAscan assay with longitudinal functional decline in a middle‐to‐old age population. We acknowledge limitations. Although the SOMAscan panel provides comprehensive insights on protein pathways, other pathways identified through lipids and metabolites can be potentially important for functional aging. Also, SOMAscan provides relative but not absolute quantification, which precludes direct comparisons with results derived by other methods. The absence of orthogonal validation of aptamers may lead to problematic protein measurements. We attempted to validate the GDF‐15 and TIMP‐1 findings using ELISA measures in FOS; however, we note the poor correlation between the ELISA and SOMAscan measures for TIMP‐1. The sample size of participants with both measures was modest; further, SOMAscan measures were conducted at examination cycle 5 (1991–1995) whereas the ELISA assays were ascertained at examination cycle 6 (1995–1998). It was also worth noting that CHS had a larger weight in the meta‐analysis owing to a larger sample size and a longer follow‐up. Moreover, the data collection, preprocessing, and standardization on physical and proteomic measures in two cohorts were conducted based on slightly different protocols. However, analyzing changes over time is on a relative scale and we applied random‐effects model in meta‐analysis to account for the possible heterogeneity across cohorts. Also, some important confounders, such as Parkinson's disease and osteoporosis, were not available, and the physical function measures were only available in two examination cycles in FOS, wherein the first cycle was 7 years after the time when protein data were available. Another limitation is that we did not include lean body mass as an outcome measure, as body composition measures were not the focus of this study. Future studies should replicate our findings in more diverse populations as well as use other assay methods for protein quantification to ensure robustness of the results.

## 5 CONCLUSIONS

The present study demonstrated that several proteins and pathways were significantly associated with longitudinal decline in gait speed and grip strength in adults from middle to older age. These discoveries may contribute to the identification of novel biomarkers and pathways that modulate physical function, which can be targeted with the goal of slowing down physical decline, delaying frailty, and preventing disability and age‐related diseases.

## AUTHOR CONTRIBUTIONS

BMP, RPT, REG, and MCO directed and supervised the project. XL and MCO configured the concept and design of study and manuscript preparation. TRA contributed to the acquisition of the data. XL and SP performed the statistical analysis. VX, R, ABN, JLS, BMP, TRA, and CW contributed to the critical revisions of the manuscript. All authors approved the final version of the manuscript and agreed to be accountable for all aspects of the work.

## CONFLICT OF INTEREST

BMP serves on the Steering Committee of the Yale Open Data Access Project funded by Johnson & Johnson. MCO serves as a consultant for Cricket Health, Inc. JLS is an employee of Vertex Pharmaceuticals at the time of publication.

## Supporting information


Appendix S1

## Data Availability

The data that support the findings of this study are available from the Cardiovascular Health Study in accordance with the policies and procedures of the study; please contact the corresponding author for details.
