# Supplementary material for: Plasma proteomic signature of decline in gait speed and grip strength
Source: Aging Cell. 2022 Nov 4;21(12):e13736. doi: 10.1111/acel.13736 (PMC9741503; doi:10.1111/acel.13736)
Supplement: Supplementary file 1 — Appendix S1 [file ACEL-21-e13736-s001.docx]

**Supplementary Material**

**3 figures, 10 tables**

**Figure S1** Flow chart of study population in CHS

**Figure S2** Flow chart of study population in FOS

**Figure S3** Distribution of age in years in Cardiovascular Heart Study (CHS) and Framingham Offspring Study (FOS)

**Table S1** Baseline characteristics of included vs. excluded subjects in CHS and FOS

**Table S2** List of proteins assayed on SOMAscan™ platform

**Table S3** Gait speed and grip strength decline in CHS and FOS

**Table S4** Significant SOMAscan proteins associated with gait speed decline by meta-analysis (fully adjusted model)

**Table S5** Significant SOMAscan proteins associated with grip strength decline by meta-analysis (fully adjusted model)

**Table S6** Significant SOMAscan proteins associated with gait speed decline by meta-analysis (exclude prevalent stroke)

**Table S7** Significant SOMAscan proteins associated with grip strength decline by meta-analysis (exclude prevalent stroke)

**Table S8** Significant SOMAscan proteins associated with gait speed decline by meta-analysis (adjusted for age × time interaction)

**Table S9** Significant SOMAscan proteins associated with grip strength decline by meta-analysis (adjusted for age × time interaction)

**Table S10** Top pathways associated with gait speed and grip strength decline using Reactome database.


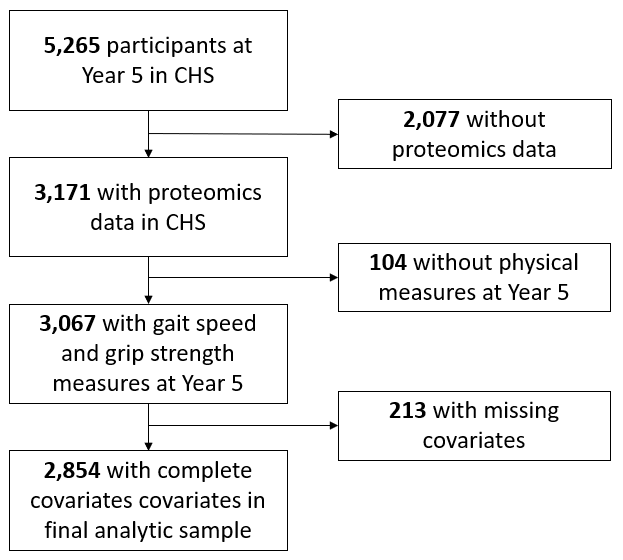


**Figure S1** Flow chart of study population in Cardiovascular Heart Study (CHS)


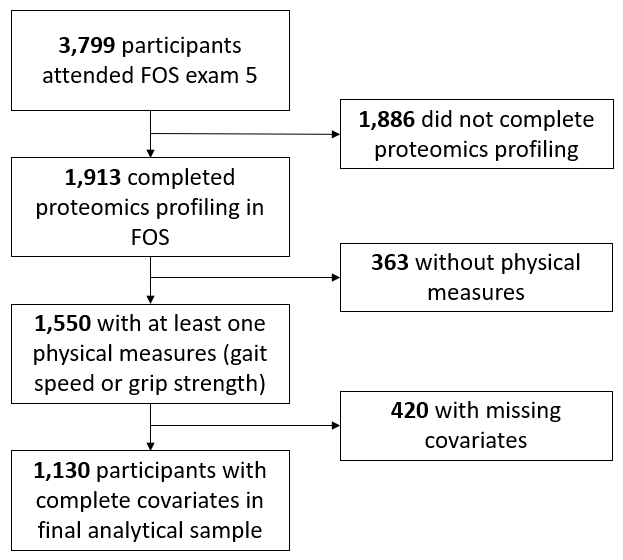


**Figure S2** Flow chart of study population in Framingham Offspring Study (FOS)


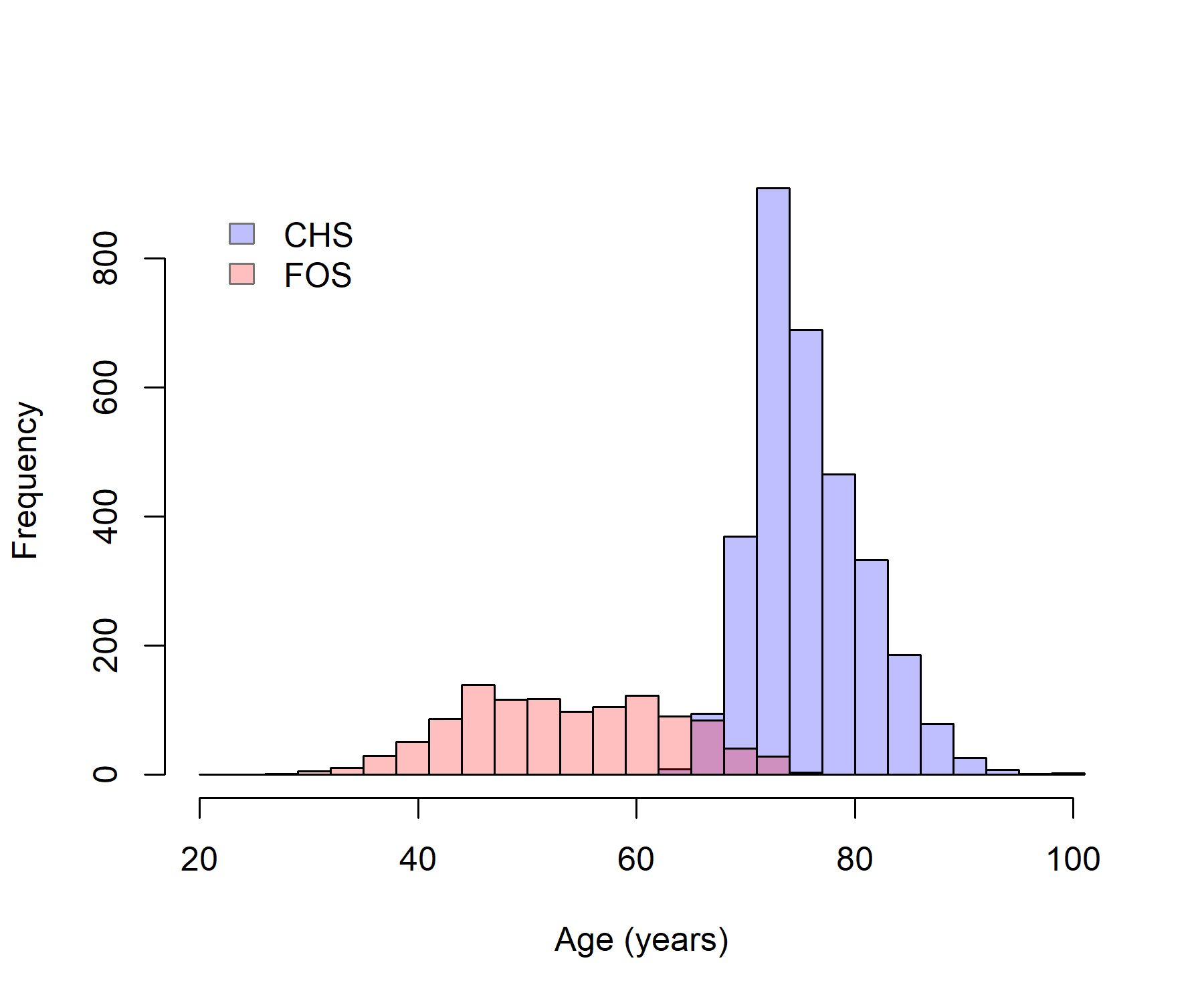


**Figure S3** Distribution of age in years in Cardiovascular Heart Study (CHS)

and Framingham Offspring Study (FOS)

**Table S1** Baseline characteristics of included vs. excluded subjects in CHS and FOS

| Variables | CHS | | | FOS | | |
| --- | --- | --- | --- | --- | --- | --- |
|  | Subjects included  (n=2,854) | Subjects excluded due to missingness  (n=317) | P-value | Subjects included  (n=1,130) | Subjects excluded due to missingness  (n=783) | P-value |
| Age, years | 76.3 (5) | 76 (5.4) | 0.466 | 54.2 (9.5) | 56.2 (10.5) | < 0.001 |
| Sex, female | 1717 (60.2) | 210 (66.2) | 0.041 | 617 (54.6) | 407 (52.0) | 0.258 |
| Race |  |  |  |  |  |  |
| Black | 419 (14.7) | 86 (27.1) |  | NA | NA | NA |
| White/other | 2,435 (85.3) | 231 (72.9) | < 0.001 |  |  |  |
| Clinic |  |  |  |  |  |  |
| Bowman gray | 734 (25.7) | 73 (23.0) |  | NA | NA | NA |
| Davis | 717 (25.1) | 115 (36.3) |  |  |  |  |
| Hopkins | 627 (22.0) | 46 (14.5) |  |  |  |  |
| Pittsburgh | 776 (27.2) | 83 (26.2) | < 0.001 |  |  |  |
| Education, years | 12.6 (2.8) | 11.9 (3.3) | < 0.001 | 14.2 (2.5) | 13.7 (2.8) | 0.001 |
| Height, cm | 164.3 (9.5) | 163.8 (9.1) | 0.400 | 167.5 (9.4) | 167.7 (9.4) | 0.349 |
| Weight, kg | 71.9 (14.1) | 73.2 (15.6) | 0.126 | 77.1 (16.1) | 77.7 (17.4) | 0.456 |
| BMI, kg/m^2^ | 26.6 (4.5) | 27.4 (5.2) | 0.002 | 27.4 (4.9) | 27.5 (5.3) | 0.681 |
| Smoking status |  |  |  |  |  |  |
| Current | 264 (9.3) | 35 (11.0) | 0.565 | 199 (17.6) | 171 (21.9) |  |
| Former | 1271 (44.5) | 136 (42.9) |  | 576 (51.0) | 388 (49.6) |  |
| Never | 1,319 (46.2) | 146 (46.1) |  | 355 (31.4) | 223 (28.5) | 0.056 |
| SBP, mmHg | 135.4 (21.2) | 138.1 (20.8) | 0.030 | 125.2 (18.6) | 128.2 (19.5) | < 0.001 |
| DBP, mmHg | 71.1 (10.9) | 72.3 (12.3) | 0.060 | 74.3 (10.1) | 74.7 (10.2) | 0.261 |
| Gait speed, m/s | 0.9 (0.2) | 0.9 (0.3) | 0.001 | 1.2 (0.3) | 1.3 (0.3) | 0.198 |
| Grip strength, kg | 28 (9.8) | 27.7 (10.2) | 0.676 | 32.8 (13.0) | 32.2 (13.2) | 0.541 |
| eGFR, mL/min/1.73m^2^ | 67.2 (17.4) | 67.2 (17.7) | 0.994 | 90.0 (19.4) | 87.4 (21.3) | 0.023 |
| 3MSE. points | 91.6 (7.7) | 88.8 (10.8) | < 0.001 | NA | NA | NA |
| Diabetes status |  |  |  |  |  |  |
| Normal | 2,190 (76.7) | 234 (73.8) | 0.417 | 1068 (94.5) | 695 (88.8) | < 0.001 |
| Diabetes | 385 (13.5) | 51 (16.1) |  | 62 (5.5) | 88 (11.2) |  |
| Impaired fasting glycemia | 279 (9.8) | 32 (10.1) |  | NA | NA | NA |
| CVD | 598 (21.0) | 84 (26.5) | 0.027 | 55 (4.9) | 68 (8.7) | 0.001 |
| Atrial fibrillation | 141 (4.9) | 15 (4.7) | 0.979 | 11 (1.0) | 17 (2.2) | 0.032 |
| COPD | 330 (11.6) | 25 (11.7) | 1.000 | NA | NA | NA |
| Cancer | 117 (4.1) | 13 (4.5) | 0.856 | 191 (16.9) | 179 (22.9) | 0.001 |
| Arthritis | 1,266 (44.4) | 167 (66.3) |  | NA | NA | NA |
| Hypertension Medication | 1342 (47.0) | 168 (53.2) | 0.044 | 187 (16.5) | 191 (24.7) | < 0.001 |

Note: Data was mean (standard deviation) or n (%) and compared using Mann-Whitney U or chi-square test.

Abbreviation: CHS, Cardiovascular Health Study; FOS, Framingham Offspring Study.

**Table S2** List of proteins assayed on SOMAscan™ platform

| SeqID | SomaId | UniProt | Target | TargetFullName |
| --- | --- | --- | --- | --- |
| 10336-3 | SL019100 | Q9UNE7 | CHIP | E3 ubiquitin-protein ligase CHIP |
| 10339-48 | SL001731 | P09104 | NSE | Gamma-enolase |
| 10342-55 | SL019096 | Q8N2W9 | PIAS4 | E3 SUMO-protein ligase PIAS4 |
| 10344-334 | SL005173 | Q13651 | IL-10 Ra | Interleukin-10 receptor subunit alpha |
| 10346-5 | SL007221 | P40763 | STAT3 | Signal transducer and activator of transcription 3 |
| 10356-21 | SL000455 | P05412 | c-Jun | Transcription factor AP-1 |
| 10361-25 | SL014684 | P00973 | OAS1 | 2'-5'-oligoadenylate synthase 1 |
| 10362-35 | SL002565 | P01106 | c-Myc | Myc proto-oncogene protein |
| 10363-13 | SL004097 | P84022 | SMAD3 | Mothers against decapentaplegic homolog 3 |
| 10364-6 | SL004101 | Q15796 | SMAD2 | Mothers against decapentaplegic homolog 2 |
| 10365-132 | SL005184 | P29460, Q9NPF7 | IL-23 | Interleukin-23 |
| 10366-11 | SL001728 | P16234 | PDGFRA | Platelet-derived growth factor receptor alpha |
| 10367-62 | SL001716 | P29459, P29460 | IL-12 | Interleukin-12 |
| 10370-21 | SL004396 | P42224 | STAT1 | Signal transducer and activator of transcription 1-alpha/beta |
| 10372-18 | SL007229 | P42226 | STAT6 | Signal transducer and activator of transcription 6 |
| 10990-21 | SL017128 | Q5S007 | LRRK2 | Leucine-rich repeat serine/threonine-protein kinase 2 |
| 11067-13 | SL001791 | P02818 | Osteocalcin | Osteocalcin |
| 11071-1 | SL000481 | P05113 | IL-5 | Interleukin-5 |
| 11089-7 | SL000459 | P01876 P01877 | IgA | Immunoglobulin A |
| 11094-104 | SL007361 | Q05315 | LPPL | Galectin-10 |
| 11096-57 | SL018921 | Q9Y5N5 | HEMK2 | HemK methyltransferase family member 2 |
| 11098-1 | SL007953 | O00764 | PDXK | Pyridoxal kinase |
| 11101-18 | SL004489 | O00206 | TLR4 | Toll-like receptor 4 |
| 11102-22 | SL012561 | Q9BYZ8 | REG4 | Regenerating islet-derived protein 4 |
| 11103-24 | SL000448 | P04792 | HSP 27 | Heat shock protein beta-1 |
| 11104-13 | SL003340 | P36222 | YKL-40 | Chitinase-3-like protein 1 |
| 11105-171 | SL003650 | P06733 | Alpha enolase | Alpha-enolase |
| 11510-31 | SL005699 | O14791 | Apo L1 | Apolipoprotein L1 |
| 11513-92 | SL002722 | P28907 | CD38 | ADP-ribosyl cyclase/cyclic ADP-ribose hydrolase 1 |
| 11514-196 | SL004557 | P13987 | CD59 | CD59 glycoprotein |
| 11516-7 | SL006131 | P07148 | FABPL | Fatty acid-binding protein, liver |
| 13088-397 | SL000137 | P35070 | BTC | Betacellulin |
| 13089-6 | SL003349 | Q16665 | HIF-1a | Hypoxia-inducible factor 1-alpha |
| 13090-17 | SL004827 | P06703 | S100A6 | Protein S100-A6 |
| 13093-6 | SL005430 | Q8WVN6 | SECTM1 | Secreted and transmembrane protein 1 |
| 13094-75 | SL018509 | Q9BXY4 | RSPO3 | R-spondin-3 |
| 13095-51 | SL005357 | P05451 | PSP | Lithostathine-1-alpha |
| 13097-11 | SL003774 | Q92843 | Apoptosis regulator Bcl-W | Bcl-2-like protein 2 |
| 13098-93 | SL003320 | O43915 | VEGF-D | Vascular endothelial growth factor D |
| 13101-60 | SL007631 | Q9BQB4 | SOST | Sclerostin |
| 13102-1 | SL012769 | Q96BQ1 | FAM3D | Protein FAM3D |
| 13103-125 | SL009328 | P0DML2 P0DML3 | CSH | Chorionic somatomammotropin hormone |
| 13104-32 | SL008614 | P98172 | EFNB1 | Ephrin-B1 |
| 13105-7 | SL007311 | P60880 | SNP25 | Synaptosomal-associated protein 25 |
| 13107-9 | SL008402 | O95274 | LYPD3 | Ly6/PLAUR domain-containing protein 3 |
| 13109-82 | SL008810 | Q7Z3B1 | NEGR1 | Neuronal growth regulator 1 |
| 13111-79 | SL007223 | P41182 | BCL6 | B-cell lymphoma 6 protein |
| 13112-179 | SL009349 | Q12841 | FSTL1 | Follistatin-related protein 1 |
| 13113-7 | SL002688 | P10451 | Osteopontin | Osteopontin |
| 13114-50 | SL006230 | P51884 | Lumican | Lumican |
| 13116-25 | SL008931 | Q8N6Q3 | CD177 | CD177 antigen |
| 13118-5 | SL011888 | Q9H4F8 | SMOC1 | SPARC-related modular calcium-binding protein 1 |
| 13119-26 | SL004467 | Q9UK55 | protein Z inhibitor | Protein Z-dependent protease inhibitor |
| 13122-19 | SL008360 | O43155 | FLRT2 | Leucine-rich repeat transmembrane protein FLRT2 |
| 13123-3 | SL008372 | Q9NZU0 | FLRT3 | Leucine-rich repeat transmembrane protein FLRT3 |
| 13124-20 | SL018587 | Q6UXK2 | ISLR2 | Immunoglobulin superfamily containing leucine-rich repeat protein 2 |
| 13125-45 | SL000616 | P04004 | Vitronectin | Vitronectin |
| 13126-52 | SL008631 | Q02487 | DSC2 | Desmocollin-2 |
| 13129-40 | SL007070 | P01130 | LDLR | Low-density lipoprotein receptor |
| 13130-150 | SL007272 | P52789 | HXK2 | Hexokinase-2 |
| 13131-5 | SL011180 | P19367 | HXK1 | Hexokinase-1 |
| 13132-14 | SL008522 | Q13591 | SEM5A | Semaphorin-5A |
| 13133-73 | SL007033 | Q8N2S1 | LTBP4 | Latent-transforming growth factor beta-binding protein 4 |
| 14114-18 | SL019019 | Q8IYJ0 | PIANP | PILR alpha-associated neural protein |
| 14115-34 | SL002782 | P35318 | Adrenomedullin | Adrenomedullin |
| 14116-129 | SL004821 | P26447 | S100A4 | Protein S100-A4 |
| 14120-2 | SL018629 | Q68DV7 | RNF43 | E3 ubiquitin-protein ligase RNF43 |
| 14121-24 | SL004159 | Q9UBN6 | TRAIL R4 | Tumor necrosis factor receptor superfamily member 10D |
| 14122-132 | SL018971 | Q9ULT6 | ZNRF3 | E3 ubiquitin-protein ligase ZNRF3 |
| 14123-34 | SL017424 | Q9H7M9 | GI24 | V-type immunoglobulin domain-containing suppressor of T-cell activation |
| 14124-6 | SL004843 | O43921 | Ephrin-A2 | Ephrin-A2 |
| 14127-240 | SL001880 | P01574 | IFN-b | Interferon beta |
| 14128-121 | SL017106 | P01566 | IFN10 | Interferon alpha-10 |
| 14129-1 | SL019978 | P01567 | IFNA7 | Interferon alpha-7 |
| 14131-37 | SL007274 | P52799 | EFNB2 | Ephrin-B2 |
| 14132-21 | SL020171 | Q9UM44 | HHLA2 | HERV-H LTR-associating protein 2 |
| 14133-93 | SL000145 | P27930 | IL-1 sRII | Interleukin-1 receptor type 2 |
| 14134-49 | SL015046 | Q86SJ2 | AMGO2 | Amphoterin-induced protein 2 |
| 14135-3 | SL020172 | Q9HBX9 | RXFP1 | Relaxin receptor 1 |
| 14136-234 | SL007696 | Q9NPY3 | C1QR1 | Complement component C1q receptor |
| 14139-16 | SL007828 | Q8WWG1 | NRG4 | Neuregulin-4 |
| 14143-8 | SL014983 | Q16778 | H2B2E | Histone H2B type 2-E |
| 14144-3 | SL019979 | Q7L7L0 | H2A3 | Histone H2A type 3 |
| 14146-92 | SL008158 | P68431 | H31 | Histone H3.1 |
| 14149-9 | SL005179 | Q9NZH7 | IL-1F8 | Interleukin-36 beta |
| 14150-7 | SL005177 | Q9UHA7 | IL-1F6 | Interleukin-36 alpha |
| 14151-4 | SL015510 | P05161 | UCRP | Ubiquitin-like protein ISG15 |
| 14153-8 | SL004139 | P52797 | Ephrin-A3 | Ephrin-A3 |
| 14156-33 | SL004815 | P31946 | 14-3-3 protein beta/alpha | 14-3-3 protein beta/alpha |
| 14157-21 | SL004984 | P62258 | 14-3-3E | 14-3-3 protein epsilon |
| 14158-17 | SL003848 | P08758 | Annexin V | Annexin A5 |
| 14583-49 | SL004357 | O14793 | Myostatin | Growth/differentiation factor 8 |
| 2182-54 | SL000318 | P0C0L4 P0C0L5 | C4b | Complement C4b |
| 2190-55 | SL002662 | P03951 | Coagulation Factor XI | Coagulation Factor XI |
| 2192-63 | SL003168 | Q9Y4X3 | CTACK | C-C motif chemokine 27 |
| 2201-17 | SL000403 | P39060 | Endostatin | Endostatin |
| 2211-9 | SL000591 | P01033 | TIMP-1 | Metalloproteinase inhibitor 1 |
| 2212-69 | SL000053 | P00750 | tPA | Tissue-type plasminogen activator |
| 2247-20 | SL004332 | P58294 | EG-VEGF | Prokineticin-1 |
| 2278-61 | SL000592 | P16035 | TIMP-2 | Metalloproteinase inhibitor 2 |
| 2333-72 | SL000584 | P01137 | TGF-b1 | Transforming growth factor beta-1 |
| 2381-52 | SL000319 | P01031 | C5 | Complement C5 |
| 2418-55 | SL000276 | P02649 | Apo E | Apolipoprotein E |
| 2421-7 | SL003951 | P23560 | BDNF | Brain-derived neurotrophic factor |
| 2429-27 | SL000324 | P07357,P07358,P07360 | C8 | Complement component C8 |
| 2431-17 | SL000345 | P08311 | Cathepsin G | Cathepsin G |
| 2436-49 | SL004016 | Q9H2A7 | CXCL16, soluble | C-X-C motif chemokine 16 |
| 2441-2 | SL004333 | O15520 | FGF-10 | Fibroblast growth factor 10 |
| 2443-10 | SL004342 | P55075 | FGF-8B | Fibroblast growth factor 8 isoform B |
| 2447-7 | SL004066 | Q9NZK7 | GIIE | Group IIE secretory phospholipase A2 |
| 2449-1 | SL004182 | P39877 | GV | Calcium-dependent phospholipase A2 |
| 2468-62 | SL000133 | P78556 | MIP-3a | C-C motif chemokine 20 |
| 2474-54 | SL000573 | P02743 | SAP | Serum amyloid P-component |
| 2475-1 | SL004010 | P10721 | SCF sR | Mast/stem cell growth factor receptor Kit |
| 2480-58 | SL003043 | P35625 | TIMP-3 | Metalloproteinase inhibitor 3 |
| 2500-2 | SL004643 | Q9Y264 | Angiopoietin-4 | Angiopoietin-4 |
| 2505-49 | SL004144 | O60609 | GFRa-3 | GDNF family receptor alpha-3 |
| 2514-65 | SL004142 | Q15768 | Ephrin-B3 | Ephrin-B3 |
| 2515-14 | SL004143 | O00451 | GFRa-2 | GDNF family receptor alpha-2 |
| 2516-57 | SL003193 | O00585 | 6Ckine | C-C motif chemokine 21 |
| 2524-56 | SL003280 | P09429 | HMG-1 | High mobility group protein B1 |
| 2558-51 | SL000300 | P01189 | b-Endorphin | Beta-endorphin |
| 2567-5 | SL003328 | P05156 | Factor I | Complement factor I |
| 2571-12 | SL000045 | P17936 | IGFBP-3 | Insulin-like growth factor-binding protein 3 |
| 2578-67 | SL000038 | P13500 | MCP-1 | C-C motif chemokine 2 |
| 2579-17 | SL000527 | P14780 | MMP-9 | Matrix metalloproteinase-9 |
| 2580-83 | SL001796 | P05164 | Myeloperoxidase | Myeloperoxidase |
| 2585-2 | SL000546 | P01236 | PRL | Prolactin |
| 2590-69 | SL006114 | Q01973 | ROR1 | Inactive tyrosine-protein kinase transmembrane receptor ROR1 |
| 2597-8 | SL000002 | P15692 | VEGF | Vascular endothelial growth factor A |
| 2599-51 | SL003735 | P41273 | 4-1BB ligand | Tumor necrosis factor ligand superfamily member 9 |
| 2602-2 | SL001996 | O15123 | Angiopoietin-2 | Angiopoietin-2 |
| 2605-49 | SL004180 | P28908 | CD30 | Tumor necrosis factor receptor superfamily member 8 |
| 2607-54 | SL004692 | O75462 Q9UBD9 | CLF-1/CLC Complex | Cytokine receptor-like factor 1:Cardiotrophin-like cytokine factor 1 Complex |
| 2609-59 | SL001777 | P01034 | Cystatin C | Cystatin-C |
| 2611-72 | SL004136 | Q06418 | Dtk | Tyrosine-protein kinase receptor TYRO3 |
| 2612-5 | SL004759 | P55010 | eIF-5 | Eukaryotic translation initiation factor 5 |
| 2614-28 | SL004140 | P52798 | Ephrin-A4 | Ephrin-A4 |
| 2615-60 | SL004141 | P52803 | Ephrin-A5 | Ephrin-A5 |
| 2616-23 | SL000009 | P04626 | ERBB2 | Receptor tyrosine-protein kinase erbB-2 |
| 2617-56 | SL002519 | P21860 | ERBB3 | Receptor tyrosine-protein kinase erbB-3 |
| 2618-10 | SL003803 | Q15303 | ERBB4 | Receptor tyrosine-protein kinase erbB-4 |
| 2619-72 | SL001890 | P09758 | GA733-1 protein | Tumor-associated calcium signal transducer 2 |
| 2620-4 | SL003872 | P40189 | gp130, soluble | Interleukin-6 receptor subunit beta |
| 2622-18 | SL004751 | P30519 | HO-2 | Heme oxygenase 2 |
| 2625-53 | SL000453 | P07900 | HSP 90a | Hsp90alpha |
| 2631-50 | SL004147 | Q08334 | IL-10 Rb | Interleukin-10 receptor subunit beta |
| 2632-5 | SL004148 | P42701 | IL-12 Rb1 | Interleukin-12 receptor subunit beta-1 |
| 2633-52 | SL004149 | P78552 | IL-13 Ra1 | Interleukin-13 receptor subunit alpha-1 |
| 2634-2 | SL003307 | P31785 | IL-2 sRg | Cytokine receptor common subunit gamma |
| 2635-61 | SL004646 | Q6UX15 | Layilin | Layilin |
| 2636-10 | SL000509 | P36941 | Lymphotoxin b R | Tumor necrosis factor receptor superfamily member 3 |
| 2637-77 | SL004579 | P22897 | Macrophage mannose receptor | Macrophage mannose receptor 1 |
| 2640-3 | SL004637 | Q04912 | MSP R | Macrophage-stimulating protein receptor |
| 2642-4 | SL004760 | P68402 | PAFAH beta subunit | Platelet-activating factor acetylhydrolase IB subunit beta |
| 2643-57 | SL004183 | P22223 | P-Cadherin | Cadherin-3 |
| 2644-11 | SL000551 | P17252 | PKC-A | Protein kinase C alpha type |
| 2645-54 | SL000557 | Q05513 | PKC-Z | Protein kinase C zeta type |
| 2647-66 | SL003648 | P50395 | Rab GDP dissociation inhibitor beta | Rab GDP dissociation inhibitor beta |
| 2649-77 | SL003178 | P32942 | sICAM-3 | Intercellular adhesion molecule 3 |
| 2652-15 | SL002506 | Q03405 | suPAR | Urokinase plasminogen activator surface receptor |
| 2654-19 | SL001992 | P19438 | TNF sR-I | Tumor necrosis factor receptor superfamily member 1A |
| 2658-27 | SL004639 | Q16288 | TrkC | NT-3 growth factor receptor |
| 2665-26 | SL004672 | Q02223 | BCMA | Tumor necrosis factor receptor superfamily member 17 |
| 2666-53 | SL004081 | P07585 | Bone proteoglycan II | Decorin |
| 2668-70 | SL000337 | P07384 P04632 | Calpain I | Calpain I |
| 2677-1 | SL002644 | P00533 | ERBB1 | Epidermal growth factor receptor |
| 2681-23 | SL000441 | P14210 | HGF | Hepatocyte growth factor |
| 2682-68 | SL000450 | P10809 | HSP 60 | 60 kDa heat shock protein, mitochondrial |
| 2683-1 | SL000456 | P01024 | iC3b | Complement C3b, inactivated |
| 2687-2 | SL001947 | Q16674 | MIA | Melanoma-derived growth regulatory protein |
| 2692-74 | SL002528 | P14555 | NPS-PLA2 | Phospholipase A2, membrane associated |
| 2693-20 | SL000530 | P13725 | OSM | Oncostatin-M |
| 2695-25 | SL001721 | P16284 | PECAM-1 | Platelet endothelial cell adhesion molecule |
| 2696-87 | SL004685 | O60542 | Persephin | Persephin |
| 2697-7 | SL003041 | P02776 | PF-4 | Platelet factor 4 |
| 2700-56 | SL000049 | P07225 | Protein S | Vitamin K-dependent protein S |
| 2704-74 | SL004364 | O14836 | TACI | Tumor necrosis factor receptor superfamily member 13B |
| 2705-5 | SL003197 | O15444 | TECK | C-C motif chemokine 25 |
| 2706-69 | SL000590 | P05543 | Thyroxine-Binding Globulin | Thyroxine-binding globulin |
| 2708-54 | SL004326 | Q9UNG2 | TNFSF18 | Tumor necrosis factor ligand superfamily member 18 |
| 2714-78 | SL003334 | Q12904 | EMAP-2 | Endothelial monocyte-activating polypeptide 2 |
| 2719-3 | SL004591 | Q99062 | G-CSF-R | Granulocyte colony-stimulating factor receptor |
| 2723-9 | SL005178 | Q9NZH6 | IL-1F7 | Interleukin-37 |
| 2728-62 | SL003198 | P24821 | Tenascin | Tenascin |
| 2730-58 | SL005199 | Q29983 | MICA | MHC class I polypeptide-related sequence A |
| 2731-29 | SL000528 | P16435 | NADPH-P450 Oxidoreductase | NADPH--cytochrome P450 reductase |
| 2732-58 | SL005235 | Q9H9S0 | NANOG | Homeobox protein NANOG |
| 2734-49 | SL005206 | O95944 | NKp44 | Natural cytotoxicity triggering receptor 2 |
| 2737-22 | SL005236 | P48745 | NovH | Protein NOV homolog |
| 2741-22 | SL005217 | O43699 | Siglec-6 | Sialic acid-binding Ig-like lectin 6 |
| 2742-68 | SL005218 | Q9Y286 | Siglec-7 | Sialic acid-binding Ig-like lectin 7 |
| 2743-5 | SL005220 | Q15465 | Sonic Hedgehog | Sonic hedgehog protein |
| 2744-57 | SL000467 | P01857 | IgG | Immunoglobulin G |
| 2747-3 | SL005229 | Q9BZM4 | ULBP-3 | NKG2D ligand 3 |
| 2750-3 | SL000019 | P02647 | Apo A-I | Apolipoprotein A-I |
| 2752-62 | SL004329 | P43026 | BMP-14 | Growth/differentiation factor 5 |
| 2753-2 | SL000309 | P02745 P02746 P02747 | C1q | Complement C1q subcomponent |
| 2754-50 | SL000312 | P01024 | C3 | Complement C3 |
| 2760-2 | SL004752 | O95990 | DRR1 | Protein FAM107A |
| 2761-49 | SL004336 | O76093 | FGF-18 | Fibroblast growth factor 18 |
| 2763-66 | SL004338 | Q9NP95 | FGF-20 | Fibroblast growth factor 20 |
| 2765-4 | SL021043 | O95390 O14793 | GDF-11/8 | Growth/differentiation factor 11/8 |
| 2770-51 | SL003176 | P22362 | I-309 | C-C motif chemokine 1 |
| 2771-35 | SL000462 | P08833 | IGFBP-1 | Insulin-like growth factor-binding protein 1 |
| 2773-50 | SL001717 | P22301 | IL-10 | Interleukin-10 |
| 2774-10 | SL000474 | Q14005 | IL-16 | Interleukin-16 |
| 2778-10 | SL004347 | Q9GZX6 | IL-22 | Interleukin-22 |
| 2780-35 | SL000496 | P02788 | Lactoferrin | Lactotransferrin |
| 2781-63 | SL004356 | Q8NHW4 | LAG-1 | C-C motif chemokine 4-like |
| 2783-18 | SL004355 | P16619 | LD78-beta | C-C motif chemokine 3-like 1 |
| 2788-55 | SL000524 | P08254 | MMP-3 | Stromelysin-1 |
| 2789-26 | SL000525 | P09237 | MMP-7 | Matrilysin |
| 2790-54 | SL003191 | P02775 | NAP-2 | Neutrophil-activating peptide 2 |
| 2794-60 | SL000581 | P00441 | SOD | Superoxide dismutase [Cu-Zn] |
| 2796-62 | SL000424 | P02671 P02675 P02679 | Fibrinogen | Fibrinogen |
| 2797-56 | SL000020 | P04114 | Apo B | Apolipoprotein B |
| 2805-6 | SL004415 | Q9BYF1 | ACE2 | Angiotensin-converting enzyme 2 |
| 2806-49 | SL004128 | P36896 | Activin RIB | Activin receptor type-1B |
| 2809-25 | SL004625 | O75173 | ADAMTS-4 | A disintegrin and metalloproteinase with thrombospondin motifs 4 |
| 2811-27 | SL001995 | Q15389 | Angiopoietin-1 | Angiopoietin-1 |
| 2813-11 | SL006924 | O00253 | ART | Agouti-related protein |
| 2816-50 | SL001902 | P50895 | BCAM | Basal Cell Adhesion Molecule |
| 2819-23 | SL002081 | P33151 | Cadherin-5 | Cadherin-5 |
| 2823-7 | SL004704 | Q86VX2 | COMMD7 | COMM domain-containing protein 7 |
| 2826-53 | SL004644 | Q92838 | EDA | Ectodysplasin-A, secreted form |
| 2827-23 | SL000427 | P78423 | Fractalkine/CX3CL-1 | Fractalkine |
| 2828-82 | SL004645 | O43278 | HAI-1 | Kunitz-type protease inhibitor 1 |
| 2829-19 | SL006915 | Q8NEV9 Q14213 | IL-27 | Interleukin-27 |
| 2831-29 | SL002763 | Q9UBX7 | Kallikrein 11 | Kallikrein-11 |
| 2833-20 | SL003800 | Q9Y5K2 | Kallikrein 4 | Kallikrein-4 |
| 2834-54 | SL003915 | O60259 | kallikrein 8 | Kallikrein-8 |
| 2835-1 | SL004301 | P12956 | Ku70 | X-ray repair cross-complementing protein 6 |
| 2836-68 | SL000695 | P80188 | Lipocalin 2 | Neutrophil gelatinase-associated lipocalin |
| 2837-3 | SL000134 | P08581 | Met | Hepatocyte growth factor receptor |
| 2838-53 | SL003332 | Q9ULZ9 | MMP-17 | Matrix metalloproteinase-17 |
| 2839-2 | SL004649 | P23510 | OX40 Ligand | Tumor necrosis factor ligand superfamily member 4 |
| 2843-13 | SL001897 | O43291 | SPINT2 | Kunitz-type protease inhibitor 2 |
| 2844-53 | SL003199 | P35590 | sTie-1 | Tyrosine-protein kinase receptor Tie-1, soluble |
| 2846-24 | SL000605 | P62979 | Ubiquitin+1 | Ubiquitin+1, truncated mutation for UbB |
| 2849-49 | SL004768 | P55008 | AIF1 | Allograft inflammatory factor 1 |
| 2851-63 | SL000320 | P01031 | C5a | C5a anaphylatoxin |
| 2853-68 | SL006911 | O14757 | CHK1 | Serine/threonine-protein kinase Chk1 |
| 2855-49 | SL000409 | P27361 | ERK-1 | Mitogen-activated protein kinase 3 |
| 2859-69 | SL004723 | Q9BY41 | HDAC8 | Histone deacetylase 8 |
| 2860-19 | SL004718 | P52292 | Karyopherin-a2 | Importin subunit alpha-1 |
| 2864-2 | SL003793 | Q02750 | MEK1 | Dual specificity mitogen-activated protein kinase kinase 1 |
| 2870-29 | SL004009 | P63000 | RAC1 | Ras-related C3 botulinum toxin substrate 1 |
| 2871-73 | SL006922 | Q06609 | RAD51 | DNA repair protein RAD51 homolog 1 |
| 2875-15 | SL006923 | P20226 | TBP | TATA-box-binding protein |
| 2876-74 | SL004305 | P11387 | Topoisomerase I | DNA topoisomerase 1 |
| 2877-3 | SL004306 | P63279 | UBC9 | SUMO-conjugating enzyme UBC9 |
| 2878-66 | SL006372 | P07947 | YES | Tyrosine-protein kinase Yes |
| 2879-9 | SL000248 | P01011 | a1-Antichymotrypsin | Alpha-1-antichymotrypsin |
| 2888-49 | SL000323 | P10643 | C7 | Complement component C7 |
| 2889-37 | SL002783 | Q16619 | Cardiotrophin-1 | Cardiotrophin-1 |
| 2890-59 | SL003303 | Q9NRJ3 | CCL28 | C-C motif chemokine 28 |
| 2900-53 | SL003329 | Q16627 | HCC-1 | C-C motif chemokine 14 |
| 2906-55 | SL000480 | P05112 | IL-4 | Interleukin-4 |
| 2911-27 | SL002621 | P21741 | Midkine | Midkine |
| 2913-1 | SL003302 | P55773 | MPIF-1 | C-C motif chemokine 23 |
| 2925-9 | SL000006 | P05121 | PAI-1 | Plasminogen activator inhibitor 1 |
| 2939-10 | SL004670 | Q5T4W7 | Artemin | Artemin |
| 2942-50 | SL000396 | P99999 | Cytochrome c | Cytochrome c |
| 2943-5 | SL000398 | P08684 | Cytochrome P450 3A4 | Cytochrome P450 3A4 |
| 2944-66 | SL005156 | P41271 | DAN | Neuroblastoma suppressor of tumorigenicity 1 |
| 2946-52 | SL003327 | P00746 | Factor D | Complement factor D |
| 2948-58 | SL005168 | P10912 | Growth hormone receptor | Growth hormone receptor |
| 2949-6 | SL004067 | O15496 | GX | Group 10 secretory phospholipase A2 |
| 2950-57 | SL005171 | P22692 | IGFBP-4 | Insulin-like growth factor-binding protein 4 |
| 2952-75 | SL000047 | P05019 | IGF-I | Insulin-like growth factor I |
| 2953-31 | SL000506 | P01215, P01229 | Luteinizing hormone | Luteinizing hormone |
| 2960-66 | SL003192 | P27918 | Properdin | Properdin |
| 2961-1 | SL000048 | P04070 | Protein C | Vitamin K-dependent protein C |
| 2962-50 | SL002561 | P12272 | PTHrP | Parathyroid hormone-related protein |
| 2966-65 | SL004362 | Q9Y240 | SCGF-beta | Stem cell growth factor-beta |
| 2967-8 | SL001720 | P19320 | VCAM-1 | Vascular cell adhesion protein 1 |
| 2968-61 | SL004686 | O95150 | TNFSF15 | Tumor necrosis factor ligand superfamily member 15 |
| 2970-60 | SL000136 | P15514 | AREG | Amphiregulin |
| 2972-57 | SL004078 | P18075 | BMP-7 | Bone morphogenetic protein 7 |
| 2973-15 | SL000668 | P16671 | CD36 ANTIGEN | Platelet glycoprotein 4 |
| 2974-61 | SL004855 | Q12860 | contactin-1 | Contactin-1 |
| 2975-19 | SL002655 | P29279 | CTGF | Connective tissue growth factor |
| 2976-58 | SL004856 | Q02413 | Desmoglein-1 | Desmoglein-1 |
| 2977-7 | SL004872 | Q9UNE0 | EDAR | Tumor necrosis factor receptor superfamily member EDAR |
| 2979-8 | SL003169 | P42830 | ENA-78 | C-X-C motif chemokine 5 |
| 2982-82 | SL005165 | P56470 | Galectin-4 | Galectin-4 |
| 2985-35 | SL003173 | P09341 | Gro-a | Growth-regulated alpha protein |
| 2987-37 | SL005256 | P16403 | Histone H1.2 | Histone H1.2 |
| 2991-9 | SL001997 | P14778 | IL-1 sRI | Interleukin-1 receptor type 1 |
| 2992-59 | SL004850 | Q96F46 | IL-17 sR | Interleukin-17 receptor A |
| 2993-1 | SL004861 | O95256 | IL-18 Rb | Interleukin-18 receptor accessory protein |
| 2994-71 | SL004875 | Q9HB29 | IL-1Rrp2 | Interleukin-1 receptor-like 2 |
| 2997-8 | SL005193 | P57087 | JAM-B | Junctional adhesion molecule B |
| 2998-53 | SL005194 | Q9BX67 | JAM-C | Junctional adhesion molecule C |
| 2999-6 | SL005196 | Q13449 | LSAMP | Limbic system-associated membrane protein |
| 3000-66 | SL004516 | P11226 | MBL | Mannose-binding protein C |
| 3003-29 | SL005205 | O14931 | NKp30 | Natural cytotoxicity triggering receptor 3 |
| 3004-67 | SL004862 | Q9BQ51 | PD-L2 | Programmed cell death 1 ligand 2 |
| 3005-5 | SL004939 | P18031 | PTP-1B | Tyrosine-protein phosphatase non-receptor type 1 |
| 3007-7 | SL005219 | Q9Y336 | Siglec-9 | Sialic acid-binding Ig-like lectin 9 |
| 3009-3 | SL005059 | Q03167 | TGF-b R III | Transforming growth factor beta receptor type 3 |
| 3010-53 | SL004687 | Q969D9 | TSLP | Thymic stromal lymphopoietin |
| 3022-4 | SL000384 | P16410 | CTLA-4 | Cytotoxic T-lymphocyte protein 4 |
| 3024-18 | SL000250 | P08697 | a2-Antiplasmin | Alpha-2-antiplasmin |
| 3025-50 | SL000004 | P09038 | bFGF | Fibroblast growth factor 2 |
| 3026-5 | SL000338 | P20810 | Calpastatin | Calpastatin |
| 3028-36 | SL003301 | P55773 | Ck-b-8-1 | Ck-beta-8-1 |
| 3029-52 | SL005157 | Q9NNX6 | DC-SIGN | CD209 antigen |
| 3030-3 | SL005158 | Q9H2X3 | DC-SIGNR | C-type lectin domain family 4 member M |
| 3032-11 | SL000428 | P01215, P01225 | FSH | Follicle stimulating hormone |
| 3033-57 | SL005164 | P05162 | Galectin-2 | Galectin-2 |
| 3034-1 | SL004271 | P14136 | GFAP | Glial fibrillary acidic protein |
| 3035-80 | SL004354 | Q9UHD0 | IL-19 | Interleukin-19 |
| 3037-62 | SL001795 | P01584 | IL-1b | Interleukin-1 beta |
| 3038-9 | SL003326 | O14625 | I-TAC | C-X-C motif chemokine 11 |
| 3040-59 | SL000519 | P10147 | MIP-1a | C-C motif chemokine 3 |
| 3041-55 | SL008416 | Q9UBG0 | MRC2 | C-type mannose receptor 2 |
| 3042-7 | SL000164 | P02144 | Myoglobin | Myoglobin |
| 3043-49 | SL000532 | P09486 | ON | SPARC |
| 3044-3 | SL003323 | P55774 | PARC | C-C motif chemokine 18 |
| 3045-72 | SL002704 | P21246 | PTN | Pleiotrophin |
| 3046-31 | SL004260 | Q9HD89 | resistin | Resistin |
| 3049-61 | SL000603 | P07477 | Trypsin | Trypsin-1 |
| 3050-7 | SL000017 | P04275 | vWF | von Willebrand factor |
| 3052-8 | SL000633 | P48023 | Fas ligand, soluble | Tumor necrosis factor ligand superfamily member 6, soluble form |
| 3054-3 | SL000437 | P00738 | Haptoglobin, Mixed Type | Haptoglobin |
| 3055-54 | SL003308 | P24394 | IL-4 sR | Interleukin-4 receptor subunit alpha |
| 3057-55 | SL004689 | O95388 | WISP-1 | WNT1-inducible-signaling pathway protein 1 |
| 3059-50 | SL004327 | Q9Y275 | BAFF | Tumor necrosis factor ligand superfamily member 13B |
| 3060-43 | SL000325 | P02748 | C9 | Complement component C9 |
| 3065-65 | SL004339 | P12034 | FGF-5 | Fibroblast growth factor 5 |
| 3066-12 | SL003744 | P17931 | Galectin-3 | Galectin-3 |
| 3067-67 | SL005234 | O60383 | GDF-9 | Growth/differentiation factor 9 |
| 3069-52 | SL000468 | P01871 | IgM | Immunoglobulin M |
| 3070-1 | SL000478 | P60568 | IL-2 | Interleukin-2 |
| 3072-4 | SL001718 | P35225 | IL-13 | Interleukin-13 |
| 3073-51 | SL002508 | O95998 | IL-18 BPa | Interleukin-18-binding protein |
| 3074-6 | SL003309 | P18428 | LBP | Lipopolysaccharide-binding protein |
| 3077-66 | SL003324 | P00742 | Coagulation Factor Xa | Coagulation factor Xa |
| 3078-1 | SL002640 | P49763 | PlGF | Placenta growth factor |
| 3079-62 | SL005152 | Q99969 | TIG2 | Retinoic acid receptor responder protein 2 |
| 3081-70 | SL005227 | Q9BZM6 | ULBP-1 | NKG2D ligand 1 |
| 3082-9 | SL005228 | Q9BZM5 | ULBP-2 | NKG2D ligand 2 |
| 3083-71 | SL005233 | Q9HAV5 | XEDAR | Tumor necrosis factor receptor superfamily member 27 |
| 3091-70 | SL004771 | O14965 | Aurora kinase A | Aurora kinase A |
| 3115-64 | SL006918 | P28482 | MK01 | Mitogen-activated protein kinase 1 |
| 3122-6 | SL003733 | Q9NR28 | SMAC | Diablo homolog, mitochondrial |
| 3132-1 | SL004486 | P49767 | VEGF-C | Vascular endothelial growth factor C |
| 3143-3 | SL002524 | P01730 | sCD4 | T-cell surface glycoprotein CD4 |
| 3151-6 | SL003305 | P01589 | IL-2 sRa | Interleukin-2 receptor subunit alpha |
| 3152-57 | SL001800 | P20333 | TNF sR-II | Tumor necrosis factor receptor superfamily member 1B |
| 3166-92 | SL005215 | P20138 | Siglec-3 | Myeloid cell surface antigen CD33 |
| 3168-8 | SL004626 | Q9UNA0 | ADAMTS-5 | A disintegrin and metalloproteinase with thrombospondin motifs 5 |
| 3169-70 | SL010368 | P35475 | IDUA | Alpha-L-iduronidase |
| 3170-6 | SL007261 | P50579 | AMPM2 | Methionine aminopeptidase 2 |
| 3171-57 | SL004469 | P05067 | amyloid precursor protein | Amyloid beta A4 protein |
| 3172-28 | SL008956 | P15848 | ARSB | Arylsulfatase B |
| 3173-49 | SL008611 | Q02083 | ASAHL | N-acylethanolamine-hydrolyzing acid amidase |
| 3174-2 | SL008421 | Q9UHI8 | ATS1 | A disintegrin and metalloproteinase with thrombospondin motifs 1 |
| 3175-51 | SL006610 | Q76LX8 | ATS13 | A disintegrin and metalloproteinase with thrombospondin motifs 13 |
| 3178-5 | SL007280 | P53634 | CATC | Dipeptidyl peptidase 1 |
| 3179-51 | SL009213 | P10619 | Cathepsin A | Lysosomal protective protein |
| 3181-50 | SL004673 | P25774 | Cathepsin S | Cathepsin S |
| 3182-38 | SL010371 | P49961 | CD39 | Ectonucleoside triphosphate diphosphohydrolase 1 |
| 3184-25 | SL000358 | P08709 | Coagulation Factor VII | Coagulation factor VII |
| 3186-2 | SL002525 | P06681 | C2 | Complement C2 |
| 3187-52 | SL007284 | P54108 | CRIS3 | Cysteine-rich secretory protein 3 |
| 3189-61 | SL010372 | P98073 | Enterokinase | Enteropeptidase |
| 3191-50 | SL012783 | Q96NZ8 | WFKN1 | WAP, kazal, immunoglobulin, kunitz and NTR domain-containing protein 1 |
| 3192-3 | SL002695 | Q96KP4 | Glutamate carboxypeptidase | Cytosolic non-specific dipeptidase |
| 3194-36 | SL008759 | Q9HCN6 | GPVI | Platelet glycoprotein VI |
| 3195-50 | SL000678 | P22749 | Granulysin | Granulysin |
| 3196-6 | SL008023 | P10915 | HPLN1 | Hyaluronan and proteoglycan link protein 1 |
| 3197-70 | SL007122 | P14735 | IDE | Insulin-degrading enzyme |
| 3198-4 | SL008639 | P22304 | IDS | Iduronate 2-sulfatase |
| 3199-54 | SL003916 | Q9UKR0 | kallikrein 12 | Kallikrein-12 |
| 3201-49 | SL003863 | Q9Y337 | kallikrein 5 | Kallikrein-5 |
| 3202-28 | SL010393 | Q8NCW0 | KREM2 | Kremen protein 2 |
| 3204-2 | SL007100 | P09960 | LKHA4 | Leukotriene A-4 hydrolase |
| 3206-4 | SL008904 | Q9Y5Y7 | LYVE1 | Lymphatic vessel endothelial hyaluronic acid receptor 1 |
| 3209-69 | SL005437 | Q9NQ76 | MEPE | Matrix extracellular phosphoglycoprotein |
| 3210-1 | SL010374 | P53582 | METAP1 | Methionine aminopeptidase 1 |
| 3212-30 | SL010375 | Q9NR71 | ASAH2 | Neutral ceramidase |
| 3213-65 | SL000640 | P14543 | Nidogen | Nidogen-1 |
| 3216-2 | SL005797 | P01833 | PIGR | Polymeric immunoglobulin receptor |
| 3220-40 | SL010378 | P07949 | RET | Proto-oncogene tyrosine-protein kinase receptor Ret |
| 3221-54 | SL003770 | Q8N474 | SARP-2 | Secreted frizzled-related protein 1 |
| 3222-11 | SL010379 | Q14563 | Semaphorin 3A | Semaphorin-3A |
| 3232-28 | SL004118 | P13686 | TrATPase | Tartrate-resistant acid phosphatase type 5 |
| 3234-23 | SL010390 | Q76M96 | URB | Coiled-coil domain-containing protein 80 |
| 3235-50 | SL010391 | Q8TEU8 | WFKN2 | WAP, Kazal, immunoglobulin, Kunitz and NTR domain-containing protein 2 |
| 3280-49 | SL004661 | P16112 | Aggrecan | Aggrecan core protein |
| 3283-21 | SL006544 | Q15582 | BGH3 | Transforming growth factor-beta-induced protein ig-h3 |
| 3284-75 | SL007804 | P21810 | BGN | Biglycan |
| 3285-23 | SL000310 | P00736 | C1r | Complement C1r subcomponent |
| 3290-50 | SL008773 | Q6YHK3 | CD109 | CD109 antigen |
| 3291-30 | SL002706 | P06734 | CD23 | Low affinity immunoglobulin epsilon Fc receptor |
| 3292-75 | SL010450 | P09326 | CD48 | CD48 antigen |
| 3293-2 | SL006108 | O43866 | CD5L | CD5 antigen-like |
| 3294-55 | SL010451 | P0CG37 | CFC1 | Cryptic protein |
| 3296-92 | SL008623 | Q02246 | CNTN2 | Contactin-2 |
| 3298-52 | SL010454 | Q8IWV2 | Contactin-4 | Contactin-4 |
| 3299-29 | SL010455 | O94779 | Contactin-5 | Contactin-5 |
| 3302-58 | SL007049 | O76096 | CYTF | Cystatin-F |
| 3305-6 | SL010457 | Q9NR61 | DLL4 | Delta-like protein 4 |
| 3309-2 | SL010459 | P12318 | FCG2A | Low affinity immunoglobulin gamma Fc region receptor II-a |
| 3310-62 | SL010460 | P31994 | FCG2B | Low affinity immunoglobulin gamma Fc region receptor II-b |
| 3311-27 | SL008609 | O75015 | FCG3B | Low affinity immunoglobulin gamma Fc region receptor III-B |
| 3312-64 | SL010461 | P12314 | FCGR1 | High affinity immunoglobulin gamma Fc receptor I |
| 3313-21 | SL006542 | Q15485 | FCN2 | Ficolin-2 |
| 3314-74 | SL004858 | P56159 | GFRa-1 | GDNF family receptor alpha-1 |
| 3315-15 | SL010463 | Q8N158 | GPC2 | Glypican-2 |
| 3316-58 | SL004466 | P05546 | Heparin cofactor II | Heparin cofactor 2 |
| 3317-33 | SL004860 | O43464 | HTRA2 | Serine protease HTRA2, mitochondrial |
| 3320-49 | SL005087 | Q16270 | IGFBP-7 | Insulin-like growth factor-binding protein 7 |
| 3321-2 | SL007385 | Q13007 | IL24 | Interleukin-24 |
| 3322-52 | SL010464 | Q6UXM1 | LRIG3 | Leucine-rich repeats and immunoglobulin-like domains protein 3 |
| 3323-37 | SL004610 | Q14114 | LRP8 | Low-density lipoprotein receptor-related protein 8 |
| 3324-51 | SL007674 | Q9HBG7 | LY9 | T-lymphocyte surface antigen Ly-9 |
| 3325-2 | SL010465 | O00339 | MATN2 | Matrilin-2 |
| 3326-58 | SL004805 | Q9BY67 | Nectin-like protein 2 | Cell adhesion molecule 1 |
| 3327-27 | SL007673 | Q9HB63 | NET4 | Netrin-4 |
| 3329-14 | SL004515 | O75594 | PGRP-S | Peptidoglycan recognition protein 1 |
| 3331-8 | SL010468 | Q6NW40 | RGMB | RGM domain family member B |
| 3332-57 | SL010469 | Q6ZVN8 | RGM-C | Hemojuvelin |
| 3336-50 | SL001998 | P10646 | TFPI | Tissue factor pathway inhibitor |
| 3339-33 | SL007206 | P35442 | TSP2 | Thrombospondin-2 |
| 3340-53 | SL007207 | P35443 | TSP4 | Thrombospondin-4 |
| 3341-33 | SL006892 | P00519 | ABL1 | Tyrosine-protein kinase ABL1 |
| 3343-1 | SL005574 | Q03154 | Aminoacylase-1 | Aminoacylase-1 |
| 3344-60 | SL000272 | P01008 | Antithrombin III | Antithrombin-III |
| 3346-72 | SL010520 | Q96GD4 | AURKB | Aurora kinase B |
| 3347-9 | SL002792 | P25098 | BARK1 | beta-adrenergic receptor kinase 1 |
| 3348-49 | SL003994 | P13497 | BMP-1 | Bone morphogenetic protein 1 |
| 3350-53 | SL010491 | Q9UQM7 | CAMK2A | Calcium/calmodulin-dependent protein kinase type II subunit alpha |
| 3351-1 | SL010492 | Q13554 | CAMK2B | Calcium/calmodulin-dependent protein kinase type II subunit beta |
| 3352-80 | SL010288 | P23280 | Carbonic anhydrase 6 | Carbonic anhydrase 6 |
| 3356-50 | SL004868 | P43166 | Carbonic anhydrase VII | Carbonic anhydrase 7 |
| 3357-67 | SL010495 | P24941 P20248 | CDK2/cyclin A | Cyclin-dependent kinase 2:Cyclin-A2 complex |
| 3358-51 | SL010496 | Q00535 Q15078 | CDK5/p35 | Cyclin-dependent kinase 5:Cyclin-dependent kinase 5 activator 1 complex |
| 3359-11 | SL010522 | P49336 P24863 | CDK8/cyclin C | Cyclin-dependent kinase 8:Cyclin-C complex |
| 3361-26 | SL010610 | Q9UJ71 | CLC4K | C-type lectin domain family 4 member K |
| 3362-61 | SL009400 | Q9BU40 | CRDL1 | Chordin-like protein 1 |
| 3363-31 | SL004781 | P41240 | CSK | Tyrosine-protein kinase CSK |
| 3364-76 | SL006910 | O60911 | Cathepsin V | Cathepsin L2 |
| 3365-7 | SL010612 | Q9UBT3 | Dkk-4 | Dickkopf-related protein 4 |
| 3366-51 | SL006550 | Q16610 | ECM1 | Extracellular matrix protein 1 |
| 3367-8 | SL006777 | Q9UGM5 | FETUB | Fetuin-B |
| 3373-5 | SL004654 | P20718 | Granzyme H | Granzyme H |
| 3374-49 | SL010499 | P08631 | HCK | Tyrosine-protein kinase HCK |
| 3376-49 | SL010613 | Q8NFM7 | IL-17 RD | Interleukin-17 receptor D |
| 3378-49 | SL000064 | P49862 | Kallikrein 7 | Kallikrein-7 |
| 3379-29 | SL007228 | P41743 | KPCI | Protein kinase C iota type |
| 3381-24 | SL010500 | P07948 | LYNB | Tyrosine-protein kinase Lyn, isoform B |
| 3387-1 | SL010508 | O75914 | PAK3 | Serine/threonine-protein kinase PAK 3 |
| 3388-58 | SL010510 | Q9P286 | PAK7 | Serine/threonine-protein kinase PAK 5 |
| 3389-7 | SL000550 | P05154 | PCI | Plasma serine protease inhibitor |
| 3390-72 | SL010512 | P42336 P27986 | PIK3CA/PIK3R1 | PIK3CA/PIK3R1 |
| 3391-10 | SL007250 | P48736 | PK3CG | Phosphatidylinositol 4,5-bisphosphate 3-kinase catalytic subunit gamma isoform |
| 3392-68 | SL003767 | Q9Y243 | PKB gamma | Protein kinase B gamma |
| 3394-81 | SL005258 | P53350 | PLK-1 | Serine/threonine-protein kinase PLK1 |
| 3396-54 | SL000565 | P00797 | Renin | Renin |
| 3397-7 | SL010616 | Q06124 | SHP-2 | Tyrosine-protein phosphatase non-receptor type 11 |
| 3399-31 | SL007560 | Q8WWQ8 | STAB2 | Stabilin-2 |
| 3400-49 | SL007747 | Q9UHD2 | TBK1 | Serine/threonine-protein kinase TBK1 |
| 3401-8 | SL005261 | P17706 | TCPTP | Tyrosine-protein phosphatase non-receptor type 2 |
| 3403-1 | SL010617 | P20231 | TPSB2 | Tryptase beta-2 |
| 3404-51 | SL010619 | Q9NRR2 | TPSG1 | Tryptase gamma |
| 3405-6 | SL010529 | Q9Y3C8 | UFC1 | Ubiquitin-fold modifier-conjugating enzyme 1 |
| 3412-7 | SL000104 | P10415 | Bcl-2 | Apoptosis regulator Bcl-2 |
| 3413-50 | SL003703 | Q16548 | BFL1 | Bcl-2-related protein A1 |
| 3414-40 | SL006374 | P51813 | BMX | Cytoplasmic tyrosine-protein kinase BMX |
| 3415-61 | SL004660 | P21815 | BSP | Bone sialoprotein 2 |
| 3418-12 | SL010490 | Q8IU85 | CAMK1D | Calcium/calmodulin-dependent protein kinase type 1D |
| 3419-49 | SL010493 | Q13557 | CAMK2D | Calcium/calmodulin-dependent protein kinase type II subunit delta |
| 3420-21 | SL004869 | Q8N1Q1 | Carbonic anhydrase XIII | Carbonic anhydrase 13 |
| 3421-54 | SL004635 | P32971 | CD30 Ligand | Tumor necrosis factor ligand superfamily member 8 |
| 3422-4 | SL010494 | P06493 P14635 | CDK1/cyclin B | Cyclin-dependent kinase 1:G2/mitotic-specific cyclin-B1 complex |
| 3423-59 | SL005250 | P23946 | Chymase | Chymase |
| 3427-63 | SL007324 | P68400 | CSK21 | Casein kinase II subunit alpha |
| 3431-54 | SL004137 | P21709 | EphA1 | Ephrin type-A receptor 1 |
| 3432-21 | SL010498 | P29320 | EPHA3 | Ephrin type-A receptor 3 |
| 3434-34 | SL010349 | P02751 | FN1.3 | Fibronectin Fragment 3 |
| 3435-53 | SL010348 | P02751 | FN1.4 | Fibronectin Fragment 4 |
| 3437-80 | SL004636 | P36888 | Flt-3 | Receptor-type tyrosine-protein kinase FLT3 |
| 3438-10 | SL009324 | O95633 | FSTL3 | Follistatin-related protein 3 |
| 3440-7 | SL004298 | P12544 | granzyme A | Granzyme A |
| 3441-64 | SL003723 | P49840 | GSK-3 alpha | Glycogen synthase kinase-3 alpha |
| 3445-53 | SL004151 | Q13261 | IL-15 Ra | Interleukin-15 receptor subunit alpha |
| 3447-64 | SL000039 | P10145 | IL-8 | Interleukin-8 |
| 3448-13 | SL004125 | P06213 | IR | Insulin receptor |
| 3449-58 | SL004876 | P29622 | Kallistatin | Kallistatin |
| 3450-4 | SL001797 | Q92876 | Kallikrein 6 | Kallikrein-6 |
| 3452-17 | SL006916 | P06239 | LCK | Tyrosine-protein kinase Lck |
| 3453-87 | SL006917 | P07948 | LYN | Tyrosine-protein kinase Lyn |
| 3457-57 | SL005084 | Q15063 | Periostin | Periostin |
| 3459-49 | SL004155 | P09619 | PDGF Rb | Platelet-derived growth factor receptor beta |
| 3461-58 | SL009089 | Q96GW7 | PGCB | Brevican core protein |
| 3466-8 | SL010513 | P17612 | PRKACA | cAMP-dependent protein kinase catalytic subunit alpha |
| 3469-74 | SL010515 | P51812 | RPS6KA3 | Ribosomal protein S6 kinase alpha-3 |
| 3470-1 | SL001945 | P16581 | sE-Selectin | E-selectin |
| 3471-49 | SL010517 | O75716 | STK16 | Serine/threonine-protein kinase 16 |
| 3472-40 | SL000582 | O15392 | Survivin | Baculoviral IAP repeat-containing protein 5 |
| 3473-78 | SL005224 | P40238 | Thrombopoietin Receptor | Thrombopoietin Receptor |
| 3474-19 | SL002705 | P07996 | Thrombospondin-1 | Thrombospondin-1 |
| 3477-63 | SL005225 | P04629 | TrkA | High affinity nerve growth factor receptor |
| 3480-7 | SL008122 | P51452 | DUS3 | Dual specificity protein phosphatase 3 |
| 3481-87 | SL011073 | Q9NQW7 | XPNPEP1 | Xaa-Pro aminopeptidase 1 |
| 3484-60 | SL000271 | P01019 | Angiotensinogen | Angiotensinogen |
| 3485-28 | SL000283 | P61769 | b2-Microglobulin | Beta-2-microglobulin |
| 3486-58 | SL000299 | P05230 | b-ECGF | Fibroblast growth factor 1 |
| 3487-32 | SL003167 | O43927 | BLC | C-X-C motif chemokine 13 |
| 3488-64 | SL000342 | P04040 | Catalase | Catalase |
| 3489-9 | SL004331 | P26441 | CNTF | Ciliary neurotrophic factor |
| 3494-71 | SL004335 | O60258 | FGF-17 | Fibroblast growth factor 17 |
| 3495-15 | SL003172 | P80162 | GCP-2 | C-X-C motif chemokine 6 |
| 3497-13 | SL002075 | P01563 | IFN-aA | Interferon alpha-2 |
| 3499-77 | SL004350 | Q9UHF5 | IL-17B | Interleukin-17B |
| 3503-4 | SL003179 | P56199, P05556 | Integrin a1b1 | Integrin alpha-I: beta-1 complex |
| 3504-58 | SL004536 | P81172 | LEAP-1 | Hepcidin |
| 3505-6 | SL000507 | P01374, Q06643 | Lymphotoxin a1/b2 | Lymphotoxin alpha1:beta2 |
| 3506-49 | SL000508 | P01374, Q06643 | Lymphotoxin a2/b1 | Lymphotoxin alpha2:beta1 |
| 3508-78 | SL003187 | O00626 | MDC | C-C motif chemokine 22 |
| 3516-60 | SL004712 | P48061 | SDF-1 | Stromal cell-derived factor 1 |
| 3518-54 | SL004015 | Q96IY4 | TAFI | Carboxypeptidase B2 |
| 3519-3 | SL003196 | Q92583 | TARC | C-C motif chemokine 17 |
| 3520-58 | SL000089 | P10600 | TGF-b3 | Transforming growth factor beta-3 |
| 3521-16 | SL000589 | P01215 P01222 | TSH | Thyroid Stimulating Hormone |
| 3522-57 | SL000615 | P01282 | Vasoactive Intestinal Peptide | Vasoactive Intestinal Peptide |
| 3534-14 | SL003862 | P29965 | CD40 ligand, soluble | CD40 ligand |
| 3535-84 | SL004367 | O94907 | DKK1 | Dickkopf-related protein 1 |
| 3538-26 | SL009216 | P20711 | dopa decarboxylase | Aromatic-L-amino-acid decarboxylase |
| 3554-24 | SL004258 | Q15848 | Adiponectin | Adiponectin |
| 3580-25 | SL000249 | P01009 | a1-Antitrypsin | Alpha-1-antitrypsin |
| 3581-53 | SL000251 | P02765 | a2-HS-Glycoprotein | Alpha-2-HS-glycoprotein |
| 3583-54 | SL005392 | P15289 | Arylsulfatase A | Arylsulfatase A |
| 3585-54 | SL009341 | P35613 | BASI | Basigin |
| 3587-53 | SL007056 | O95393 | BMP10 | Bone morphogenetic protein 10 |
| 3592-4 | SL010489 | Q14012 | CAMK1 | Calcium/calmodulin-dependent protein kinase type 1 |
| 3593-72 | SL003711 | P42574 | Caspase-3 | Caspase-3 |
| 3600-2 | SL006029 | Q13231 | Chitotriosidase-1 | Chitotriosidase-1 |
| 3603-60 | SL007640 | Q9BXN2 | CLC7A | C-type lectin domain family 7 member A |
| 3605-77 | SL011049 | P48740 | MASP3 | Mannan-binding lectin serine protease 1 |
| 3607-71 | SL009412 | Q9UBP4 | DKK3 | Dickkopf-related protein 3 |
| 3616-3 | SL008504 | P15586 | GNS | N-acetylglucosamine-6-sulfatase |
| 3617-80 | SL006512 | Q04756 | HGFA | Hepatocyte growth factor activator |
| 3620-67 | SL007806 | Q8N6P7 | IL22RA1 | Interleukin-22 receptor subunit alpha-1 |
| 3622-33 | SL008909 | Q99538 | LGMN | Legumain |
| 3623-84 | SL007059 | O95711 | LY86 | Lymphocyte antigen 86 |
| 3627-71 | SL010376 | Q495T6 | MMEL2 | Membrane metallo-endopeptidase-like 1 |
| 3628-3 | SL010501 | P36507 | MP2K2 | Dual specificity mitogen-activated protein kinase kinase 2 |
| 3629-60 | SL006805 | Q9Y5S2 | MRCKB | Serine/threonine-protein kinase MRCK beta |
| 3630-27 | SL004804 | Q8N126 | Nectin-like protein 1 | Cell adhesion molecule 3 |
| 3640-14 | SL005263 | P30533 | RAP | alpha-2-macroglobulin receptor-associated protein |
| 3647-49 | SL018625 | O00206 Q9Y6Y9 | TLR4:MD-2 complex | Toll-like receptor 4:Lymphocyte antigen 96 complex |
| 3651-50 | SL003201 | P35968 | VEGF sR2 | Vascular endothelial growth factor receptor 2 |
| 3685-53 | SL010466 | Q9BY79 | MFRP | Membrane frizzled-related protein |
| 3708-62 | SL000252 | P01023 | a2-Macroglobulin | Alpha-2-macroglobulin |
| 3710-49 | SL000268 | P00747 | Angiostatin | Angiostatin |
| 3714-49 | SL000382 | P12277 P06732 | CK-MB | Creatine kinase M-type:Creatine kinase B-type heterodimer |
| 3719-2 | SL000076 | P46527 | p27Kip1 | Cyclin-dependent kinase inhibitor 1B |
| 3723-1 | SL000306 | P16860 | BNP-32 | Brain natriuretic peptide 32 |
| 3727-35 | SL011509 | P10082 | PYY | Peptide YY |
| 3728-52 | SL000570 | P09683 | Secretin | Secretin |
| 3730-81 | SL011448 | P43489 | TNR4 | Tumor necrosis factor receptor superfamily member 4 |
| 3738-54 | SL002684 | P09603 | CSF-1 | Macrophage colony-stimulating factor 1 |
| 3758-68 | SL003974 | P04070 | Activated Protein C | Activated Protein C |
| 3761-4 | SL000027 | P35354 | COX-2 | Prostaglandin G/H synthase 2 |
| 3773-15 | SL003200 | Q02763 | sTie-2 | Angiopoietin-1 receptor, soluble |
| 3795-6 | SL004642 | Q13443 | ADAM 9 | Disintegrin and metalloproteinase domain-containing protein 9 |
| 3796-79 | SL007642 | Q9BY76 | ANGL4 | Angiopoietin-related protein 4 |
| 3797-1 | SL000638 | P19022 | Cadherin-2 | Cadherin-2 |
| 3798-71 | SL005508 | Q16790 | Carbonic anhydrase 9 | Carbonic anhydrase 9 |
| 3799-11 | SL004867 | P07451 | Carbonic anhydrase III | Carbonic anhydrase 3 |
| 3800-71 | SL000377 | P12277 | CK-BB | Creatine kinase B-type |
| 3803-10 | SL008382 | P28325 | CYTD | Cystatin-D |
| 3805-16 | SL010458 | Q9NQ30 | Endocan | Endothelial cell-specific molecule 1 |
| 3806-55 | SL004844 | P54756 | EphA5 | Ephrin type-A receptor 5 |
| 3807-1 | SL007651 | Q9GZV9 | FGF23 | Fibroblast growth factor 23 |
| 3808-76 | SL003990 | P21802 | FGFR-2 | Fibroblast growth factor receptor 2 |
| 3809-1 | SL004063 | P22607 | FGFR-3 | Fibroblast growth factor receptor 3 |
| 3810-50 | SL006912 | P09769 | FGR | Tyrosine-protein kinase Fgr |
| 3813-3 | SL006913 | P06241 | FYN | Tyrosine-protein kinase Fyn |
| 3815-14 | SL007620 | Q99665 | IL-12 RB2 | Interleukin-12 receptor subunit beta-2 |
| 3817-18 | SL007358 | Q04759 | KPCT | Protein kinase C theta type |
| 3820-68 | SL010503 | P49137 | MAPK2 | MAP kinase-activated protein kinase 2 |
| 3821-28 | SL010504 | Q8IW41 | MAPK5 | MAP kinase-activated protein kinase 5 |
| 3822-54 | SL004765 | Q16644 | MAPKAPK3 | MAP kinase-activated protein kinase 3 |
| 3823-9 | SL010505 | P42679 | MATK | Megakaryocyte-associated tyrosine-protein kinase |
| 3825-18 | SL010502 | P45983 | MK08 | Mitogen-activated protein kinase 8 |
| 3827-22 | SL010509 | Q9NQU5 | PAK6 | Serine/threonine-protein kinase PAK 6 |
| 3831-21 | SL003761 | P60484 | pTEN | Phosphatidylinositol 3,4,5-trisphosphate 3-phosphatase and dual-specificity protein phosphatase PTEN |
| 3832-51 | SL010514 | Q13882 | PTK6 | Protein-tyrosine kinase 6 |
| 3835-11 | SL004492 | O60603 | TLR2 | Toll-like receptor 2 |
| 3836-51 | SL010528 | P61960 | UFM1 | Ubiquitin-fold modifier 1 |
| 3839-60 | SL011629 | O00170 | AIP | AH receptor-interacting protein |
| 3844-2 | SL004823 | P62937 | Cyclophilin A | Peptidyl-prolyl cis-trans isomerase A |
| 3845-51 | SL011530 | Q9NP97 | DLRB1 | Dynein light chain roadblock-type 1 |
| 3847-56 | SL011532 | O95571 | ETHE1 | Persulfide dioxygenase ETHE1, mitochondrial |
| 3848-14 | SL003785 | P04406 | GAPDH, liver | Glyceraldehyde-3-phosphate dehydrogenase |
| 3852-19 | SL000449 | P25685 | HSP 40 | DnaJ homolog subfamily B member 1 |
| 3853-56 | SL008102 | P40925 | MDHC | Malate dehydrogenase, cytoplasmic |
| 3854-24 | SL011631 | Q13765 | NACA | Nascent polypeptide-associated complex subunit alpha |
| 3855-56 | SL004919 | Q06830 | Peroxiredoxin-1 | Peroxiredoxin-1 |
| 3858-5 | SL008063 | P24666 | PPAC | Low molecular weight phosphotyrosine protein phosphatase |
| 3859-50 | SL010973 | P25786 | PSA1 | Proteasome subunit alpha type-1 |
| 3860-7 | SL005630 | P60900 | PSA6 | Proteasome subunit alpha type-6 |
| 3864-5 | SL011528 | P62081 | RS7 | 40S ribosomal protein S7 |
| 3866-7 | SL011529 | Q9Y3A5 | SBDS | Ribosome maturation protein SBDS |
| 3867-49 | SL011630 | Q6UXD5 | SE6L2 | Seizure 6-like protein 2 |
| 3868-8 | SL011533 | O43765 | SGTA | Small glutamine-rich tetratricopeptide repeat-containing protein alpha |
| 3872-2 | SL005679 | P13693 | TCTP | Translationally-controlled tumor protein |
| 3873-51 | SL000588 | P07202 | TMA | Thyroid peroxidase |
| 3874-8 | SL008157 | P68036 | UB2L3 | Ubiquitin-conjugating enzyme E2 L3 |
| 3875-62 | SL011549 | Q99856 | ARI3A | AT-rich interactive domain-containing protein 3A |
| 3879-50 | SL011232 | Q16543 | CDC37 | Hsp90 co-chaperone Cdc37 |
| 3881-49 | SL003753 | P63167 | DLC8 | Dynein light chain 1, cytoplasmic |
| 3887-90 | SL010927 | Q14974 | IMB1 | Importin subunit beta-1 |
| 3888-8 | SL009628 | Q9UK53 | ING1 | Inhibitor of growth protein 1 |
| 3889-64 | SL006132 | P20700 | Lamin-B1 | Lamin-B1 |
| 3890-8 | SL000493 | P07195 | LDH-H 1 | L-lactate dehydrogenase B chain |
| 3891-56 | SL009629 | O95243 | MBD4 | Methyl-CpG-binding domain protein 4 |
| 3892-21 | SL010328 | Q15648 | MED-1 | Mediator of RNA polymerase II transcription subunit 1 |
| 3893-64 | SL001905 | Q13421 | Mesothelin | Mesothelin |
| 3894-15 | SL005629 | Q9UJ70 | NAGK | N-acetyl-D-glucosamine kinase |
| 3896-5 | SL004820 | P18669 | Phosphoglycerate mutase 1 | Phosphoglycerate mutase 1 |
| 3897-61 | SL011709 | Q96GD0 | PLPP | Pyridoxal phosphate phosphatase |
| 3898-5 | SL007266 | P51665 | PSD7 | 26S proteasome non-ATPase regulatory subunit 7 |
| 3902-21 | SL008808 | P63208 | SKP1 | S-phase kinase-associated protein 1 |
| 3903-49 | SL005372 | O95219 | Sorting nexin 4 | Sorting nexin-4 |
| 3905-62 | SL008143 | P61088 | UBE2N | Ubiquitin-conjugating enzyme E2 N |
| 4122-12 | SL004119 | Q08345 | discoidin domain receptor 1 | Epithelial discoidin domain-containing receptor 1 |
| 4123-60 | SL003171 | P08620 | FGF-4 | Fibroblast growth factor 4 |
| 4124-24 | SL000451 | P0DMV8 | HSP 70 | Heat shock 70 kDa protein 1A |
| 4125-52 | SL003680 | Q15109 | sRAGE | Advanced glycosylation end product-specific receptor, soluble |
| 4126-22 | SL004511 | P17213 | BPI | Bactericidal permeability-increasing protein |
| 4127-75 | SL000322 | P13671 | C6 | Complement component C6 |
| 4128-27 | SL003104 | O00175 | Eotaxin-2 | C-C motif chemokine 24 |
| 4129-72 | SL000414 | P00751 | Factor B | Complement factor B |
| 4130-71 | SL004340 | P10767 | FGF-6 | Fibroblast growth factor 6 |
| 4131-72 | SL000426 | P02751 | Fibronectin | Fibronectin |
| 4132-27 | SL000674 | P19883 | FST | Follistatin |
| 4133-54 | SL004068 | P10144 | Granzyme B | Granzyme B |
| 4135-84 | SL000461 | P01854 | IgE | Immunoglobulin E |
| 4136-40 | SL004353 | Q8TAD2 | IL-17D | Interleukin-17D |
| 4137-57 | SL004351 | Q9H293 | IL-17E | Interleukin-25 |
| 4138-25 | SL004346 | Q9NYY1 | IL-20 | Interleukin-20 |
| 4140-3 | SL000483 | P13232 | IL-7 | Interleukin-7 |
| 4141-79 | SL003183 | P02778 | IP-10 | C-X-C motif chemokine 10 |
| 4143-74 | SL003186 | P47992 | Lymphotactin | Lymphotactin |
| 4144-13 | SL000517 | Q99616 | MCP-4 | C-C motif chemokine 13 |
| 4145-58 | SL004359 | P20783 | Neurotrophin-3 | Neurotrophin-3 |
| 4146-58 | SL004360 | P34130 | Neurotrophin-5 | Neurotrophin-4 |
| 4148-49 | SL002755 | Q13219 | PAPP-A | Pappalysin-1 |
| 4149-8 | SL000537 | P01127 | PDGF-BB | Platelet-derived growth factor subunit B |
| 4150-75 | SL000540 | P00747 | Plasmin | Plasmin |
| 4151-6 | SL000541 | P00747 | Plasminogen | Plasminogen |
| 4152-58 | SL000545 | P03952 | Prekallikrein | Plasma kallikrein |
| 4153-11 | SL018548 | P01011 | alpha-1-antichymotrypsin complex | Alpha-1-antichymotrypsin complex |
| 4154-57 | SL000560 | P16109 | P-Selectin | P-selectin |
| 4155-3 | SL003198 | P24821 | Tenascin | Tenascin |
| 4156-74 | SL000088 | P61812 | TGF-b2 | Transforming growth factor beta-2 |
| 4157-2 | SL000586 | P00734 | Thrombin | Thrombin |
| 4158-54 | SL000613 | P00749 | uPA | Urokinase-type plasminogen activator |
| 4159-130 | SL000415 | P08603 | Factor H | Complement factor H |
| 4160-49 | SL000124 | P08253 | MMP-2 | 72 kDa type IV collagenase |
| 4162-54 | SL000601 | P02787 | Transferrin | Serotransferrin |
| 4163-5 | SL002093 | P0C0S5 | Histone H2A.z | Histone H2A.z |
| 4179-57 | SL004989 | P61981 | 14-3-3 protein gamma | 14-3-3 protein gamma |
| 4184-43 | SL008378 | Q13542 | 4EBP2 | Eukaryotic translation initiation factor 4E-binding protein 2 |
| 4187-49 | SL000247 | P52209 | 6-Phosphogluconate dehydrogenase | 6-phosphogluconate dehydrogenase, decarboxylating |
| 4188-1 | SL003658 | O43488 | Aflatoxin B1 aldehyde reductase | Aflatoxin B1 aldehyde reductase member 2 |
| 4192-10 | SL008039 | P14550 | AK1A1 | Alcohol dehydrogenase [NADP(+)] |
| 4194-26 | SL011769 | Q92688 | AN32B | Acidic leucine-rich nuclear phosphoprotein 32 family member B |
| 4203-50 | SL004920 | P23528 | Cofilin-1 | Cofilin-1 |
| 4209-60 | SL004757 | Q9NP79 | DRG-1 | Vacuolar protein sorting-associated protein VTA1 homolog |
| 4212-5 | SL012188 | Q9UBC2 | EP15R | Epidermal growth factor receptor substrate 15-like 1 |
| 4217-49 | SL004795 | Q99714 | ERAB | 3-hydroxyacyl-CoA dehydrogenase type-2 |
| 4220-39 | SL005588 | P16591 | FER | Tyrosine-protein kinase Fer |
| 4224-7 | SL007022 | O60506 | HNRPQ | Heterogeneous nuclear ribonucleoprotein Q |
| 4230-1 | SL011211 | P78344 | IF4G2 | Eukaryotic translation initiation factor 4 gamma 2 |
| 4232-19 | SL003304 | P08069 | IGF-I sR | Insulin-like growth factor 1 receptor |
| 4234-8 | SL004146 | Q01638 | IL-1 R4 | Interleukin-1 receptor-like 1 |
| 4237-70 | SL011770 | Q9UIC8 | LCMT1 | Leucine carboxyl methyltransferase 1 |
| 4240-31 | SL002650 | P14618 | M2-PK | Pyruvate kinase PKM |
| 4246-40 | SL004154 | P32004 | NCAM-L1 | Neural cell adhesion molecule L1 |
| 4249-64 | SL004921 | P22392 | NDP kinase B | Nucleoside diphosphate kinase B |
| 4250-23 | SL006268 | Q9UNZ2 | NSF1C | NSFL1 cofactor p47 |
| 4254-6 | SL011768 | Q8IVD9 | NUDC3 | NudC domain-containing protein 3 |
| 4258-15 | SL008331 | Q9UQ80 | PA2G4 | Proliferation-associated protein 2G4 |
| 4261-55 | SL005493 | P27169 | paraoxonase 1 | Serum paraoxonase/arylesterase 1 |
| 4267-81 | SL011772 | O00541 | PESC | Pescadillo homolog |
| 4271-75 | SL006705 | Q99471 | PFD5 | Prefoldin subunit 5 |
| 4272-46 | SL000539 | P06744 | PHI | Glucose-6-phosphate isomerase |
| 4276-10 | SL005358 | P30086 | prostatic binding protein | Phosphatidylethanolamine-binding protein 1 |
| 4278-14 | SL004901 | P07237 | Protein disulfide-isomerase | Protein disulfide-isomerase |
| 4280-47 | SL008865 | P25787 | PSA2 | Proteasome subunit alpha type-2 |
| 4282-3 | SL005034 | P62826 | RAN | GTP-binding nuclear protein Ran |
| 4284-18 | SL011535 | Q14498 | RBM39 | RNA-binding protein 39 |
| 4292-5 | SL011202 | P54920 | SNAA | Alpha-soluble NSF attachment protein |
| 4294-16 | SL006088 | Q9NYA1 | Sphingosine kinase 1 | Sphingosine kinase 1 |
| 4297-62 | SL005115 | Q9HCB6 | Spondin-1 | Spondin-1 |
| 4301-58 | SL000057 | P04183 | Thymidine kinase | Thymidine kinase, cytosolic |
| 4304-18 | SL006698 | Q8N3X6 | transcription factor MLR1, isoform CRA_b | Ligand-dependent nuclear receptor corepressor-like protein |
| 4306-4 | SL003655 | P29401 | Transketolase | Transketolase |
| 4309-59 | SL004812 | P60174 | Triosephosphate isomerase | Triosephosphate isomerase |
| 4314-12 | SL011809 | Q9H773 | XTP3A | dCTP pyrophosphatase 1 |
| 4318-12 | SL004940 | P29350 | PTP-1C | Tyrosine-protein phosphatase non-receptor type 6 |
| 4322-28 | SL013489 | Q9BXJ7 | AMNLS | Protein amnionless |
| 4324-33 | SL008516 | P09228 | CYTT | Cystatin-SA |
| 4328-2 | SL013490 | Q9BWV1 | BOC | Brother of CDO |
| 4332-6 | SL013488 | Q9P126 | CLC1B | C-type lectin domain family 1 member B |
| 4337-49 | SL000051 | P02741 | CRP | C-reactive protein |
| 4342-10 | SL002922 | P05362 | sICAM-1 | Intercellular adhesion molecule 1 |
| 4355-13 | SL007752 | Q9UIK4 | DAPK2 | Death-associated protein kinase 2 |
| 4359-87 | SL013570 | O43781 | DYRK3 | Dual specificity tyrosine-phosphorylation-regulated kinase 3 |
| 4374-45 | SL003869 | Q99988 | MIC-1 | Growth/differentiation factor 15 |
| 4389-2 | SL007003 | O43323 | DHH | Desert hedgehog protein N-product |
| 4392-54 | SL005161 | P61328 | FGF-12 | Fibroblast growth factor 12 |
| 4393-3 | SL004334 | O43320 | FGF-16 | Fibroblast growth factor 16 |
| 4394-71 | SL014308 | P55075 | FGF-8A | Fibroblast growth factor 8 isoform A |
| 4396-54 | SL004348 | Q8IU54 | IFN-lambda 1 | Interferon lambda-1 |
| 4397-26 | SL004349 | Q8IZJ0 | IFN-lambda 2 | Interferon lambda-2 |
| 4407-10 | SL005202 | P26927 | MSP | Hepatocyte growth factor-like protein |
| 4413-3 | SL001888 | P03973 | SLPI | Antileukoproteinase |
| 4420-7 | SL009988 | O43184 | ADAM12 | Disintegrin and metalloproteinase domain-containing protein 12 |
| 4423-77 | SL003674 | Q07817 | BCL2-like 1 protein | Bcl-2-like protein 1 |
| 4429-51 | SL014130 | Q9GZX3 | CHST6 | Carbohydrate sulfotransferase 6 |
| 4430-44 | SL006713 | Q9BWP8 | Collectin Kidney 1 | Collectin-11 |
| 4435-66 | SL009045 | Q6UWV6 | ENPP7 | Ectonucleotide pyrophosphatase/phosphodiesterase family member 7 |
| 4436-1 | SL014093 | O75355 | ENTP3 | Ectonucleoside triphosphate diphosphohydrolase 3 |
| 4437-56 | SL014028 | O75356 | ENTP5 | Ectonucleoside triphosphate diphosphohydrolase 5 |
| 4440-15 | SL014088 | Q96P31 | FCRL3 | Fc receptor-like protein 3 |
| 4453-83 | SL014108 | Q86VH5 | LRRT3 | Leucine-rich repeat transmembrane neuronal protein 3 |
| 4455-89 | SL006523 | Q08431 | MFGM | Lactadherin |
| 4459-68 | SL014069 | Q16549 | PCSK7 | Proprotein convertase subtilisin/kexin type 7 |
| 4460-8 | SL006998 | O15530 | PDPK1 | 3-phosphoinositide-dependent protein kinase 1 |
| 4464-10 | SL001753 | Q9BZZ2 | Sialoadhesin | Sialoadhesin |
| 4467-49 | SL005488 | Q14515 | SPARCL1 | SPARC-like protein 1 |
| 4468-21 | SL014029 | Q9NRA0 | SPHK2 | Sphingosine kinase 2 |
| 4469-78 | SL007502 | Q7LFX5 | ST4S6 | Carbohydrate sulfotransferase 15 |
| 4471-50 | SL008945 | Q08188 | TGM3 | Protein-glutamine gamma-glutamyltransferase E |
| 4472-5 | SL004908 | P07951 | Tropomyosin 2 | Tropomyosin beta chain |
| 4474-19 | SL004070 | P62979 | Ubiquitin | Ubiquitin |
| 4476-22 | SL010519 | P43403 | ZAP70 | Tyrosine-protein kinase ZAP-70 |
| 4479-14 | SL000308 | P05155 | C1-Esterase Inhibitor | Plasma protease C1 inhibitor |
| 4480-59 | SL000314 | P01024 | C3b | Complement C3b |
| 4481-34 | SL000316 | P0C0L4, P0C0L5 | C4 | Complement C4 |
| 4482-66 | SL000321 | P01031,P13671 | C5b, 6 Complex | Complement C5b-C6 complex |
| 4487-1 | SL001691 | P21781 | FGF7 | Fibroblast growth factor 7 |
| 4493-92 | SL000470 | P20809 | IL-11 | Interleukin-11 |
| 4496-60 | SL000522 | P39900 | MMP-12 | Macrophage metalloelastase |
| 4498-62 | SL003764 | P13591 | NCAM-120 | Neural cell adhesion molecule 1, 120 kDa isoform |
| 4499-21 | SL000535 | P04085 | PDGF-AA | Platelet-derived growth factor subunit A |
| 4500-50 | SL004363 | Q9Y240 | SCGF-alpha | Stem cell growth factor-alpha |
| 4533-76 | SL012740 | Q8TE58 | ATS15 | A disintegrin and metalloproteinase with thrombospondin motifs 15 |
| 4534-10 | SL012822 | Q9GZN4 | BSSP4 | Brain-specific serine protease 4 |
| 4535-50 | SL008644 | Q10588 | BST1 | ADP-ribosyl cyclase/cyclic ADP-ribose hydrolase 2 |
| 4540-11 | SL009768 | P45973 | CBX5 | Chromobox protein homolog 5 |
| 4541-49 | SL014092 | Q4KMG0 | CDON | Cell adhesion molecule-related/down-regulated by oncogenes |
| 4542-24 | SL001896 | P10909 | Clusterin | Clusterin |
| 4543-65 | SL014048 | Q86Y22 | CONA1 | Collagen alpha-1(XXIII) chain |
| 4544-4 | SL004708 | P02775 | CTAP-III | Connective tissue-activating peptide III |
| 4545-53 | SL006197 | Q96DA6 | DnaJ homolog | Mitochondrial import inner membrane translocase subunit TIM14 |
| 4546-27 | SL008822 | Q9UHX3 | EMR2 | Adhesion G protein-coupled receptor E2 |
| 4547-59 | SL014071 | Q9NZU1 | FLRT1 | Leucine-rich repeat transmembrane protein FLRT1 |
| 4548-4 | SL005575 | P21217 | Fucosyltransferase 3 | Galactoside 3(4)-L-fucosyltransferase |
| 4549-78 | SL014008 | Q11128 | FUT5 | Alpha-(1,3)-fucosyltransferase 5 |
| 4551-72 | SL014094 | Q8IZF4 | GP114 | Adhesion G-protein coupled receptor G5 |
| 4553-65 | SL014009 | Q7Z4V5 | HDGR2 | Hepatoma-derived growth factor-related protein 2 |
| 4556-10 | SL013548 | Q6ZMJ4 | IL-34 | Interleukin-34 |
| 4557-61 | SL014111 | Q8IZU9 | KIRR3 | Kin of IRRE-like protein 3 |
| 4559-64 | SL013969 | Q16719 | KYNU | Kynureninase |
| 4562-1 | SL012457 | P58417 | NXPH1 | Neurexophilin-1 |
| 4563-61 | SL012108 | P19174 | PLCG1 | 1-phosphatidylinositol 4,5-bisphosphate phosphodiesterase gamma-1 |
| 4564-2 | SL006406 | O60486 | PLXC1 | Plexin-C1 |
| 4566-24 | SL013989 | Q6UXX9 | RSPO2 | R-spondin-2 |
| 4567-82 | SL014468 | O60880 | SH21A | SH2 domain-containing protein 1A |
| 4568-17 | SL014070 | O94991 | SLIK5 | SLIT and NTRK-like protein 5 |
| 4569-52 | SL014091 | Q96PQ0 | SORC2 | VPS10 domain-containing receptor SorCS2 |
| 4588-1 | SL011499 | P01298 | PH | Pancreatic hormone |
| 4673-13 | SL000087 | P05231 | IL-6 | Interleukin-6 |
| 4693-72 | SL008085 | P31937 | 3HIDH | 3-hydroxyisobutyrate dehydrogenase, mitochondrial |
| 4697-59 | SL001726 | P04141 | GM-CSF | Granulocyte-macrophage colony-stimulating factor |
| 4703-87 | SL000597 | P01374 | TNF-b | Lymphotoxin-alpha |
| 4706-17 | SL010830 | P11171 | 41 | Protein 4.1 |
| 4708-3 | SL004253 | P14061 | 17-beta-HSD 1 | Estradiol 17-beta-dehydrogenase 1 |
| 4712-28 | SL005361 | P05090 | Apo D | Apolipoprotein D |
| 4717-55 | SL000479 | P08700 | IL-3 | Interleukin-3 |
| 4718-5 | SL007869 | P23284 | PPIB | Peptidyl-prolyl cis-trans isomerase B |
| 4719-58 | SL003524 | P30101 | Protein disulfide isomerase A3 | Protein disulfide-isomerase A3 |
| 4721-54 | SL006119 | Q07654 | TFF3 | Trefoil factor 3 |
| 4763-31 | SL004742 | P43652 | Afamin | Afamin |
| 4769-10 | SL008590 | Q6UX06 | Olfactomedin-4 | Olfactomedin-4 |
| 4771-10 | SL012754 | Q92484 | ASM3A | Acid sphingomyelinase-like phosphodiesterase 3a |
| 4775-34 | SL005572 | P06396 | Gelsolin | Gelsolin |
| 4785-30 | SL000347 | P08185 | CBG | Corticosteroid-binding globulin |
| 4801-13 | SL007153 | P22079 | PERL | Lactoperoxidase |
| 4807-13 | SL008072 | P27658 | CO8A1 | Collagen alpha-1(VIII) chain |
| 4811-33 | SL004739 | Q14624 | ITI heavy chain H4 | Inter-alpha-trypsin inhibitor heavy chain H4 |
| 4829-43 | SL001737 | P31947 | STRATIFIN | 14-3-3 protein sigma |
| 4831-4 | SL002823 | P14151 | sL-Selectin | L-Selectin |
| 4832-75 | SL004156 | O00220 | TRAIL R1 | Tumor necrosis factor receptor superfamily member 10A |
| 4834-61 | SL002654 | P29317 | Epithelial cell kinase | Ephrin type-A receptor 2 |
| 4840-73 | SL001729 | P09919 | G-CSF | Granulocyte colony-stimulating factor |
| 4842-62 | SL000070 | P51654 | Glypican 3 | Glypican-3 |
| 4851-25 | SL000125 | P01583 | IL-1a | Interleukin-1 alpha |
| 4862-63 | SL004133 | Q13873 | BMP RII | Bone morphogenetic protein receptor type-2 |
| 4866-59 | SL004160 | Q16620 | TrkB | BDNF/NT-3 growth factors receptor |
| 4867-15 | SL003310 | P15692 | VEGF121 | Vascular endothelial growth factor A, isoform 121 |
| 4874-3 | SL000003 | P03950 | Angiogenin | Angiogenin |
| 4876-32 | SL000357 | P00740 | Coagulation Factor IX | Coagulation factor IX |
| 4878-3 | SL000360 | P00742 | Coagulation Factor X | Coagulation Factor X |
| 4880-21 | SL007756 | Q9UK05 | GDF2 | Growth/differentiation factor 2 |
| 4883-56 | SL000021 | P01308 | Insulin | Insulin |
| 4886-3 | SL000516 | P80098 | MCP-3 | C-C motif chemokine 7 |
| 4889-82 | SL009951 | O00755 | WNT7A | Protein Wnt-7a |
| 4890-10 | SL003461 | P01189 | ACTH | Corticotropin |
| 4891-50 | SL000433 | P01275 | Glucagon | Glucagon |
| 4900-8 | SL000313 | P01024 | C3a | C3a anaphylatoxin |
| 4903-72 | SL003657 | Q08209 P63098 | Calcineurin | Calcineurin |
| 4904-7 | SL003710 | P42575 | Caspase-2 | Caspase-2 |
| 4905-63 | SL004814 | Q14019 | Coactosin-like protein | Coactosin-like protein |
| 4906-35 | SL000622 | P12259 | Coagulation Factor V | Coagulation Factor V |
| 4907-56 | SL000022 | P02671 P02675 P02679 | D-dimer | D-dimer |
| 4908-6 | SL004482 | P17813 | Endoglin | Endoglin |
| 4909-68 | SL005167 | O00214 | Galectin-8 | Galectin-8 |
| 4910-21 | SL004064 | P04054 | GIB | Phospholipase A2 |
| 4911-49 | SL003643 | P09211 | Glutathione S-transferase Pi | Glutathione S-transferase P |
| 4912-17 | SL000280 | P17174 | GOT1 | Aspartate aminotransferase, cytoplasmic |
| 4913-78 | SL003300 | O15467 | HCC-4 | C-C motif chemokine 16 |
| 4914-10 | SL001766 | P01215,P01233 | HCG | Human Chorionic Gonadotropin |
| 4915-64 | SL000836 | P69905, P68871 | Hemoglobin | Hemoglobin |
| 4916-2 | SL000460 | P01880 | IgD | Immunoglobulin D |
| 4917-62 | SL003182 | P06756, P18084 | Integrin aVb5 | Integrin alpha-V: beta-5 complex |
| 4920-10 | SL000510 | P61626 | Lysozyme | Lysozyme C |
| 4922-13 | SL003189 | Q99731 | MIP-3b | C-C motif chemokine 19 |
| 4923-79 | SL005201 | P03971 | MIS | Muellerian-inhibiting factor |
| 4924-32 | SL000521 | P03956 | MMP-1 | Interstitial collagenase |
| 4925-54 | SL000523 | P45452 | MMP-13 | Collagenase 3 |
| 4929-55 | SL005102 | P04278 | SHBG | Sex hormone-binding globulin |
| 4930-21 | SL005789 | P52823 | Stanniocalcin-1 | Stanniocalcin-1 |
| 4931-59 | SL000024 | P13726 | TF | Tissue Factor |
| 4956-2 | SL000139 | O14944 | EPI | Epiregulin |
| 4959-2 | SL004925 | O95994 | AGR2 | Anterior gradient protein 2 homolog |
| 4960-72 | SL004208 | P04083 | annexin I | Annexin A1 |
| 4961-17 | SL004209 | P07355 | annexin II | Annexin A2 |
| 4962-52 | SL012538 | Q49AH0 | ARMEL | Cerebral dopamine neurotrophic factor |
| 4963-19 | SL011708 | P56211 | ARP19 | cAMP-regulated phosphoprotein 19 |
| 4964-67 | SL007729 | Q9NZ08 | ARTS1 | Endoplasmic reticulum aminopeptidase 1 |
| 4965-27 | SL005675 | P06576 | ATP synthase beta chain | ATP synthase subunit beta, mitochondrial |
| 4967-1 | SL008177 | Q07021 | C1QBP | Complement component 1 Q subcomponent-binding protein, mitochondrial |
| 4968-50 | SL008099 | P40121 | CAPG | Macrophage-capping protein |
| 4969-2 | SL004866 | P00915 | Carbonic anhydrase I | Carbonic anhydrase 1 |
| 4970-55 | SL000339 | P00918 | carbonic anhydrase II | Carbonic anhydrase 2 |
| 4971-1 | SL008380 | Q9UBR2 | CATZ | Cathepsin Z |
| 4973-18 | SL003728 | Q13489 | cIAP-2 | Baculoviral IAP repeat-containing protein 3 |
| 4976-57 | SL013240 | P46108 | CRK | Adapter molecule crk |
| 4978-54 | SL011628 | Q9UJU6 | DBNL | Drebrin-like protein |
| 4979-34 | SL008178 | Q07507 | DERM | Dermatopontin |
| 4981-6 | SL008709 | Q14574 | DSC3 | Desmocollin-3 |
| 4982-54 | SL004458 | P19957 | Elafin | Elafin |
| 4984-83 | SL006378 | P10768 | Esterase D | S-formylglutathione hydrolase |
| 4985-11 | SL005352 | Q01469 | FABPE | Fatty acid-binding protein, epidermal |
| 4986-59 | SL012248 | Q05397 | FAK1 | Focal adhesion kinase 1 |
| 4987-17 | SL010373 | P24071 | FCAR | Immunoglobulin alpha Fc receptor |
| 4988-49 | SL002036 | P22455 | FGFR4 | Fibroblast growth factor receptor 4 |
| 4989-7 | SL003341 | P02679 | Fibrinogen g-chain dimer | Fibrinogen gamma chain |
| 4990-87 | SL006460 | P07359 | GP1BA | Platelet glycoprotein Ib alpha chain |
| 4991-12 | SL012469 | P78333 | GPC5 | Glypican-5 |
| 4992-49 | SL007173 | P28799 | GRN | Granulins |
| 4993-16 | SL000670 | Q16772 | GSTA3 | Glutathione S-transferase A3 |
| 4995-16 | SL003930 | P15428 | HPG- | 15-hydroxyprostaglandin dehydrogenase [NAD(+)] |
| 4996-66 | SL006448 | P04196 | HRG | Histidine-rich glycoprotein |
| 4997-19 | SL011616 | P38919 | IF4A3 | Eukaryotic initiation factor 4A-III |
| 5000-52 | SL006522 | Q08380 | LG3BP | Galectin-3-binding protein |
| 5001-6 | SL001973 | O75556 | Mammaglobin 2 | Mammaglobin-B |
| 5002-76 | SL002646 | P50281 | MMP-14 | Matrix metalloproteinase-14 |
| 5004-69 | SL007453 | Q15759 | MK11 | Mitogen-activated protein kinase 11 |
| 5005-4 | SL007281 | P53778 | MK12 | Mitogen-activated protein kinase 12 |
| 5006-71 | SL006993 | O15264 | MK13 | Mitogen-activated protein kinase 13 |
| 5007-1 | SL006920 | Q16539 | MAPK14 | Mitogen-activated protein kinase 14 |
| 5008-51 | SL001815 | P04179 | Mn SOD | Superoxide dismutase [Mn], mitochondrial |
| 5009-11 | SL005846 | P26038 | Moesin | Moesin |
| 5011-11 | SL003685 | P43490 | PBEF | Nicotinamide phosphoribosyltransferase |
| 5012-67 | SL004296 | P00568 | Myokinase, human | Adenylate kinase isoenzyme 1 |
| 5013-2 | SL004915 | O00299 | NCC27 | Chloride intracellular channel protein 1 |
| 5014-49 | SL006091 | P16333 | NCK1 | Cytoplasmic protein NCK1 |
| 5015-15 | SL003440 | Q13093 | PAFAH | Platelet-activating factor acetylhydrolase |
| 5017-19 | SL004932 | P30044 | Peroxiredoxin-5 | Peroxiredoxin-5, mitochondrial |
| 5018-68 | SL005694 | P30041 | Peroxiredoxin-6 | Peroxiredoxin-6 |
| 5019-16 | SL002803 | P09936 | PGP9.5 | Ubiquitin carboxyl-terminal hydrolase isozyme L1 |
| 5020-50 | SL003653 | P00558 | phosphoglycerate kinase 1 | Phosphoglycerate kinase 1 |
| 5021-13 | SL004914 | Q15181 | PPase | Inorganic pyrophosphatase |
| 5023-23 | SL009792 | P30566 | PUR8 | Adenylosuccinate lyase |
| 5024-67 | SL002522 | P06400 | Rb | Retinoblastoma-associated protein |
| 5026-66 | SL008059 | P23396 | RS3 | 40S ribosomal protein S3 |
| 5028-59 | SL005764 | Q86VB7 | sCD163 | Scavenger receptor cysteine-rich type 1 protein M130 |
| 5029-3 | SL006528 | Q12884 | SEPR | Prolyl endopeptidase FAP |
| 5030-52 | SL006629 | Q8IXJ6 | SIRT2 | NAD-dependent protein deacetylase sirtuin-2 |
| 5031-10 | SL008190 | Q13813 | SPTA2 | Spectrin alpha chain, non-erythrocytic 1 |
| 5032-64 | SL009868 | Q08945 | SSRP1 | FACT complex subunit SSRP1 |
| 5033-27 | SL004737 | P09493 | Tropomyosin 1 alpha chain | Tropomyosin alpha-1 chain |
| 5034-79 | SL010388 | P07478 | Trypsin 2 | Trypsin-2 |
| 5035-7 | SL000142 | P04818 | TS | Thymidylate synthase |
| 5036-50 | SL004782 | P98066 | TSG-6 | Tumor necrosis factor-inducible gene 6 protein |
| 5060-62 | SL004852 | Q9NZQ7 | B7-H1 | Programmed cell death 1 ligand 1 |
| 5061-27 | SL004853 | O75144 | B7-H2 | ICOS ligand |
| 5062-60 | SL011100 | Q15762 | CD226 | CD226 antigen |
| 5063-12 | SL014188 | Q9BZW8 | CD244 | Natural killer cell receptor 2B4 |
| 5065-8 | SL014229 | Q01151 | CD83 | CD83 antigen |
| 5066-134 | SL014270 | Q08708 | CLM6 | CMRF35-like molecule 6 |
| 5069-9 | SL004556 | P08174 | DAF | Complement decay-accelerating factor |
| 5070-76 | SL003739 | O95407 | DcR3 | Tumor necrosis factor receptor superfamily member 6B |
| 5076-53 | SL014294 | Q5JZY3 | EPHAA | Ephrin type-A receptor 10 |
| 5078-82 | SL008414 | O15197 | EphB6 | Ephrin type-B receptor 6 |
| 5082-51 | SL004849 | Q9NP60 | IL-1 sR9 | X-linked interleukin-1 receptor accessory protein-like 2 |
| 5085-18 | SL005181 | Q9UHF4 | IL-20 Ra | Interleukin-20 receptor subunit alpha |
| 5087-5 | SL005183 | Q969J5 | IL-22BP | Interleukin-22 receptor subunit alpha-2 |
| 5088-175 | SL005185 | Q5VWK5 | IL-23 R | Interleukin-23 receptor |
| 5089-11 | SL005189 | P16871 | IL-7 Ra | Interleukin-7 receptor subunit alpha |
| 5090-49 | SL005190 | Q8NHL6 | ILT-2 | Leukocyte immunoglobulin-like receptor subfamily B member 1 |
| 5091-28 | SL005191 | Q8N423 | ILT-4 | Leukocyte immunoglobulin-like receptor subfamily B member 2 |
| 5092-51 | SL007328 | P78504 | JAG1 | Protein jagged-1 |
| 5093-47 | SL007774 | Q9Y219 | JAG2 | Protein jagged-2 |
| 5094-62 | SL009202 | Q86YT9 | JAML1 | Junctional adhesion molecule-like |
| 5095-21 | SL012698 | Q99706 | KI2L4 | Killer cell immunoglobulin-like receptor 2DL4 |
| 5096-51 | SL014269 | P43630 | KI3L2 | Killer cell immunoglobulin-like receptor 3DL2 |
| 5097-14 | SL014289 | Q14943 | KI3S1 | Killer cell immunoglobulin-like receptor 3DS1 |
| 5098-79 | SL014209 | Q9NZS2 | KLRF1 | Killer cell lectin-like receptor subfamily F member 1 |
| 5100-53 | SL005197 | Q14108 | LIMP II | Lysosome membrane protein 2 |
| 5102-55 | SL005200 | Q29980 | MICB | MHC class I polypeptide-related sequence B |
| 5103-30 | SL014288 | Q8TD46 | MO2R1 | Cell surface glycoprotein CD200 receptor 1 |
| 5105-2 | SL005208 | Q9BZR6 | Nogo Receptor | Reticulon-4 receptor |
| 5106-52 | SL007356 | Q04721 | NOTC2 | Neurogenic locus notch homolog protein 2 |
| 5107-7 | SL005703 | P46531 | Notch 1 | Neurogenic locus notch homolog protein 1 |
| 5108-72 | SL005209 | Q9UM47 | Notch-3 | Neurogenic locus notch homolog protein 3 |
| 5109-24 | SL005210 | Q92823 | Nr-CAM | Neuronal cell adhesion molecule |
| 5110-84 | SL009054 | P58400 | NRX1B | Neurexin-1-beta |
| 5111-15 | SL008728 | Q9HDB5 | NRX3B | Neurexin-3-beta |
| 5112-73 | SL014268 | P41217 | OX2G | OX-2 membrane glycoprotein |
| 5114-65 | SL005212 | P16471 | Prolactin Receptor | Prolactin receptor |
| 5116-62 | SL007680 | Q9HCK4 | ROBO2 | Roundabout homolog 2 |
| 5117-14 | SL014148 | Q96MS0 | ROBO3 | Roundabout homolog 3 |
| 5121-3 | SL014248 | Q9H3T3 | SEM6B | Semaphorin-6B |
| 5124-69 | SL005169 | Q9UMF0 | sICAM-5 | Intercellular adhesion molecule 5 |
| 5128-53 | SL014228 | Q96DU3 | SLAF6 | SLAM family member 6 |
| 5129-12 | SL005221 | Q14162 | SREC-I | Scavenger receptor class F member 1 |
| 5131-15 | SL004863 | Q9NS68 | TAJ | Tumor necrosis factor receptor superfamily member 19 |
| 5132-71 | SL005223 | Q6UWB1 | TCCR | Interleukin-27 receptor subunit alpha |
| 5133-17 | SL002078 | P37173 | TGF-b R II | TGF-beta receptor type-2 |
| 5134-52 | SL007547 | Q8TDQ0 | TIMD3 | Hepatitis A virus cellular receptor 2 |
| 5138-50 | SL004366 | Q9NP84 | TWEAKR | Tumor necrosis factor receptor superfamily member 12A |
| 5139-32 | SL005230 | O95185 | UNC5H3 | Netrin receptor UNC5C |
| 5178-5 | SL011406 | Q13946 | PDE7A | High affinity cAMP-specific 3',5'-cyclic phosphodiesterase 7A |
| 5183-53 | SL016548 | Q13131 Q9Y478 P54619 | AMPK a1b1g1 | AMP Kinase (alpha1beta1gamma1) |
| 5196-7 | SL006476 | P30419 | NMT1 | Glycylpeptide N-tetradecanoyltransferase 1 |
| 5201-50 | SL016554 | O76083 | PDE9A | High affinity cGMP-specific 3',5'-cyclic phosphodiesterase 9A |
| 5202-4 | SL007373 | Q08752 | PPID | Peptidyl-prolyl cis-trans isomerase D |
| 5204-13 | SL005308 | P61289 | PSME3 | Proteasome activator complex subunit 3 |
| 5223-59 | SL015728 | Q14397 | GCKR | Glucokinase regulatory protein |
| 5225-50 | SL016550 | P68400 P67870 | CK2-A1:B | Casein kinase II 2-alpha:2-beta heterotetramer |
| 5226-36 | SL016551 | P19784 P67870 | CK2-A2:B | Casein kinase II 2-alpha':2-beta heterotetramer |
| 5227-60 | SL006921 | Q15118 | PDK1 | [Pyruvate dehydrogenase (acetyl-transferring)] kinase isozyme 1, mitochondrial |
| 5228-25 | SL006189 | Q02241 | KIF23 | Kinesin-like protein KIF23 |
| 5229-90 | SL014735 | P20839 | IMDH1 | Inosine-5'-monophosphate dehydrogenase 1 |
| 5230-99 | SL016557 | P04035 | HMGR | 3-hydroxy-3-methylglutaryl-coenzyme A reductase |
| 5231-79 | SL012707 | Q8NBP7 | PCSK9 | Proprotein convertase subtilisin/kexin type 9 |
| 5236-2 | SL007145 | P20393 | NR1D1 | Nuclear receptor subfamily 1 group D member 1 |
| 5238-26 | SL013928 | Q9UNP9 | PPIE | Peptidyl-prolyl cis-trans isomerase E |
| 5242-37 | SL007237 | P45985 | MP2K4 | Dual specificity mitogen-activated protein kinase kinase 4 |
| 5245-40 | SL016549 | P54646 O43741 P54619 | AMPK a2b2g1 | AMP Kinase (alpha2beta2gamma1) |
| 5246-64 | SL005730 | O00408 | cGMP-stimulated PDE | cGMP-dependent 3',5'-cyclic phosphodiesterase |
| 5248-68 | SL005793 | P30405 | Cyclophilin F | Peptidyl-prolyl cis-trans isomerase F, mitochondrial |
| 5249-31 | SL016566 | O94768 | DRAK2 | Serine/threonine-protein kinase 17B |
| 5250-53 | SL010928 | P12268 | IMDH2 | Inosine-5'-monophosphate dehydrogenase 2 |
| 5252-33 | SL016555 | Q9HCR9 | PDE11 | Dual 3',5'-cyclic-AMP and -GMP phosphodiesterase 11A |
| 5253-1 | SL011400 | P54750 | PDE1A | Calcium/calmodulin-dependent 3',5'-cyclic nucleotide phosphodiesterase 1A |
| 5254-69 | SL016553 | Q14432 | PDE3A | cGMP-inhibited 3',5'-cyclic phosphodiesterase A |
| 5255-22 | SL011404 | Q08499 | PDE4D | cAMP-specific 3',5'-cyclic phosphodiesterase 4D |
| 5256-86 | SL011405 | O76074 | PDE5A | cGMP-specific 3',5'-cyclic phosphodiesterase |
| 5259-2 | SL016567 | O43318 Q15750 | TAK1-TAB1 | Mitogen-activated protein kinase kinase kinase 7:TGF-beta-activated kinase 1 and MAP3K7-binding protein 1 fusion |
| 5260-80 | SL007181 | P29597 | TYK2 | Non-receptor tyrosine-protein kinase TYK2 |
| 5261-13 | SL010488 | P42684 | ABL2 | Abelson tyrosine-protein kinase 2 |
| 5262-57 | SL014470 | O75815 | BCAR3 | Breast cancer anti-estrogen resistance protein 3 |
| 5264-65 | SL003520 | P27797 | calreticulin | Calreticulin |
| 5265-12 | SL005725 | O75791 | GRB2-related adapter protein 2 | GRB2-related adapter protein 2 |
| 5268-49 | SL003331 | P51512 | MMP-16 | Matrix metalloproteinase-16 |
| 5272-55 | SL014469 | P29353 | SHC1 | SHC-transforming protein 1 |
| 5275-28 | SL014488 | P15498 | VAV | Proto-oncogene vav |
| 5280-68 | SL016563 | Q9H1K4 | GHC2 | Mitochondrial glutamate carrier 2 |
| 5301-7 | SL000406 | P51671 | Eotaxin | Eotaxin |
| 5307-12 | SL004400 | P00740 | Coagulation Factor IXab | Coagulation factor IXab |
| 5315-22 | SL000052 | P45379 | Troponin T | Troponin T, cardiac muscle |
| 5316-54 | SL000558 | P00734 | Prothrombin | Prothrombin |
| 5335-73 | SL003647 | P08133 | annexin VI | Annexin A6 |
| 5337-64 | SL004131 | P42081 | B7-2 | T-lymphocyte activation antigen CD86 |
| 5339-49 | SL004477 | P06702 | calgranulin B | Protein S100-A9 |
| 5340-24 | SL003717 | Q92851 | Caspase-10 | Caspase-10 |
| 5343-74 | SL008703 | P16870 | CBPE | Carboxypeptidase E |
| 5345-51 | SL006675 | Q8WWK9 | CKAP2 | Cytoskeleton-associated protein 2 |
| 5346-24 | SL011808 | Q99829 | CPNE1 | Copine-1 |
| 5347-59 | SL000130 | P14635 | Cyclin B1 | G2/mitotic-specific cyclin-B1 |
| 5349-69 | SL006970 | O00548 | DLL1 | Delta-like protein 1 |
| 5350-14 | SL012881 | Q9Y625 | GPC6 | Glypican-6 |
| 5351-52 | SL004891 | P22626 | hnRNP A2/B1 | Heterogeneous nuclear ribonucleoproteins A2/B1 |
| 5352-11 | SL004145 | Q92956 | HVEM | Tumor necrosis factor receptor superfamily member 14 |
| 5353-89 | SL001990 | P18510 | IL-1Ra | Interleukin-1 receptor antagonist protein |
| 5354-11 | SL000655 | P05783 | Keratin 18 | Keratin, type I cytoskeletal 18 |
| 5355-69 | SL004648 | O43557 | LIGHT | Tumor necrosis factor ligand superfamily member 14 |
| 5357-60 | SL014113 | Q8N0W4 | NLGNX | Neuroligin-4, X-linked |
| 5358-3 | SL008574 | Q99983 | OMD | Osteomodulin |
| 5359-65 | SL002702 | P11309 | PIM1 | Serine/threonine-protein kinase pim-1 |
| 5360-9 | SL003722 | P31751 | PKB beta | RAC-beta serine/threonine-protein kinase |
| 5363-51 | SL010470 | O15041 | Semaphorin 3E | Semaphorin-3E |
| 5364-7 | SL007336 | Q01105 | SET | Protein SET |
| 5383-14 | SL004671 | Q96RJ3 | BAFF Receptor | Tumor necrosis factor receptor superfamily member 13C |
| 5392-73 | SL002731 | P25445 | Fas, soluble | Tumor necrosis factor receptor superfamily member 6 |
| 5400-52 | SL003184 | P48357 | sLeptin R | Leptin receptor, soluble |
| 5404-53 | SL004871 | O75509 | DR6 | Tumor necrosis factor receptor superfamily member 21 |
| 5410-53 | SL007295 | P55291 | CAD15 | Cadherin-15 |
| 5412-53 | SL004134 | P26842 | CD27 | CD27 antigen |
| 5430-66 | SL008967 | P78324 | SHPS1 | Tyrosine-protein phosphatase non-receptor type substrate 1 |
| 5437-63 | SL001774 | P05413 | FABP | Fatty acid-binding protein, heart |
| 5440-26 | SL004594 | P48788 | Troponin I, skeletal, fast twitch | Troponin I, fast skeletal muscle |
| 5441-67 | SL001761 | P19429 | Troponin I | Troponin I, cardiac muscle |
| 5443-62 | SL002505 | P01160 | ANP | Atrial natriuretic factor |
| 5451-1 | SL003166 | Q13740 | ALCAM | CD166 antigen |
| 5452-71 | SL008835 | P07306 | ASGR1 | Asialoglycoprotein receptor 1 |
| 5456-59 | SL006694 | Q96KN2 | CNDP1 | Beta-Ala-His dipeptidase |
| 5457-5 | SL007471 | Q5KU26 | COLEC12 | Collectin-12 |
| 5459-33 | SL010456 | P01037 | CYTN | Cystatin-SN |
| 5460-60 | SL004750 | Q9UMR2 | DEAD-box protein 19B | ATP-dependent RNA helicase DDX19B |
| 5462-62 | SL002086 | O75636 | Ficolin-3 | Ficolin-3 |
| 5463-22 | SL000658 | P54826 | GAS1 | Growth arrest-specific protein 1 |
| 5464-52 | SL003792 | P62993 | GRB2 adapter protein | Growth factor receptor-bound protein 2 |
| 5465-32 | SL014129 | O60243 | H6ST1 | Heparan-sulfate 6-O-sulfotransferase 1 |
| 5467-15 | SL000454 | P08238 | HSP 90b | Heat shock protein HSP 90-beta |
| 5468-67 | SL011068 | Q8NAC3 | IL-17 RC | Interleukin-17 receptor C |
| 5475-10 | SL000553 | P05771 | PKC-B-II | Protein kinase C beta type (splice variant beta-II) |
| 5476-66 | SL000556 | P05129 | PKC-G | Protein kinase C gamma type |
| 5478-50 | SL000158 | Q04609 | PSMA | Glutamate carboxypeptidase 2 |
| 5480-49 | SL000563 | P13501 | RANTES | C-C motif chemokine 5 |
| 5481-16 | SL013754 | P20936 | RASA1 | Ras GTPase-activating protein 1 |
| 5483-1 | SL010467 | Q96B86 | RGMA | Repulsive guidance molecule A |
| 5484-63 | SL011588 | P61247 | RS3A | 40S ribosomal protein S3a |
| 5486-73 | SL003177 | P13598 | sICAM-2 | Intercellular adhesion molecule 2 |
| 5487-7 | SL016928 | Q9NQ25 | SLAF7 | SLAM family member 7 |
| 5488-74 | SL010516 | P12931 | SRCN1 | Proto-oncogene tyrosine-protein kinase Src isoform 2 |
| 5489-18 | SL010250 | P31948 | Stress-induced-phosphoprotein 1 | Stress-induced-phosphoprotein 1 |
| 5490-53 | SL010384 | Q08629 | Testican-1 | Testican-1 |
| 5491-12 | SL010471 | Q92563 | Testican-2 | Testican-2 |
| 5493-17 | SL010524 | Q9BYP7 | WNK3 | Serine/threonine-protein kinase WNK3 |
| 5494-52 | SL009790 | P62306 | RUXF | Small nuclear ribonucleoprotein F |
| 5508-62 | SL000344 | P07339 | Cathepsin D | Cathepsin D |
| 5509-7 | SL000084 | P01133 | EGF | Epidermal growth factor |
| 5526-53 | SL004859 | Q9Y5U5 | GITR | Tumor necrosis factor receptor superfamily member 18 |
| 5532-53 | SL003060 | P11362 | bFGF-R | Fibroblast growth factor receptor 1 |
| 5534-49 | SL004157 | O14763 | TRAIL R2 | Tumor necrosis factor receptor superfamily member 10B |
| 5542-22 | SL006397 | O14786 | NRP1 | Neuropilin-1 |
| 5792-8 | SL000011 | P02771 | AFP | alpha-Fetoprotein |
| 5798-3 | SL003704 | P55957 | BID | BH3-interacting domain death agonist |
| 5801-72 | SL000305 | P01138 | b-NGF | beta-nerve growth factor |
| 5803-24 | SL003362 | P01024 | C3d | Complement C3d fragment |
| 5807-77 | SL007195 | P32970 | CD70 | CD70 antigen |
| 5810-25 | SL005155 | P13385 | Cripto | Teratocarcinoma-derived growth factor 1 |
| 5813-58 | SL000408 | P01588 | Epo | Erythropoietin |
| 5822-22 | SL004248 | P39905 | GDNF | Glial cell line-derived neurotrophic factor |
| 5825-49 | SL000458 | P15260 | IFN-g R1 | Interferon gamma receptor 1 |
| 5834-18 | SL000485 | P15248 | IL-9 | Interleukin-9 |
| 5837-49 | SL004714 | P42702 | LIF sR | Leukemia inhibitory factor receptor |
| 5843-60 | SL003542 | Q96KQ7 | NG36 | Histone-lysine N-methyltransferase EHMT2 |
| 5852-6 | SL004783 | P80511 | S100A12 | Protein S100-A12 |
| 5854-60 | SL004230 | P10636 | tau | Microtubule-associated protein tau |
| 5858-6 | SL005688 | P63104 | 14-3-3 protein zeta/delta | 14-3-3 protein zeta/delta |
| 5861-78 | SL008466 | P46952 | 3HAO | 3-hydroxyanthranilate 3,4-dioxygenase |
| 5864-10 | SL004910 | P04075 | aldolase A | Fructose-bisphosphate aldolase A |
| 5867-60 | SL008008 | P05089 | ARGI1 | Arginase-1 |
| 5870-23 | SL003700 | Q92934 | BAD | Bcl2-associated agonist of cell death |
| 5879-51 | SL009207 | Q13561 | Dynactin subunit 2 | Dynactin subunit 2 |
| 5882-34 | SL005685 | P24534 | EF-1-beta | Elongation factor 1-beta |
| 5885-55 | SL004924 | Q15056 | eIF-4H | Eukaryotic translation initiation factor 4H |
| 5888-29 | SL005687 | P63241 | eIF-5A-1 | Eukaryotic translation initiation factor 5A-1 |
| 5897-58 | SL002071 | P07492 | Gastrin-releasing peptide | Gastrin-releasing peptide |
| 5900-11 | SL009431 | P49773 | HINT1 | Histidine triad nucleotide-binding protein 1 |
| 5903-91 | SL004899 | P11142 | HSP70 protein 8 | Heat shock cognate 71 kDa protein |
| 5909-51 | SL003687 | P15531 | Nucleoside diphosphate kinase A | Nucleoside diphosphate kinase A |
| 5915-58 | SL012148 | P50542 | PEX5 | Peroxisomal targeting signal 1 receptor |
| 5918-5 | SL008176 | Q06323 | PSME1 | Proteasome activator complex subunit 1 |
| 5927-4 | SL004690 | O95389 | WISP-3 | WNT1-inducible-signaling pathway protein 3 |
| 5934-1 | SL000420 | P02794 P02792 | Ferritin | Ferritin |
| 5936-53 | SL002517 | P01375 | TNF-a | Tumor necrosis factor |
| 5939-42 | SL004365 | O43508 | TWEAK | Tumor necrosis factor ligand superfamily member 12 |
| 5947-90 | SL000598 | P40225 | Tpo | Thrombopoietin |
| 5954-62 | SL003970 | P01270 | PTH | Parathyroid hormone |
| 5957-30 | SL011510 | P61278 | Somatostatin-28 | Somatostatin-28 |
| 6151-18 | SL007242 | P46734 | MP2K3 | Dual specificity mitogen-activated protein kinase kinase 3 |
| 6641-60 | SL017289 | P0CG47 | PolyUbiquitin K48 | PolyUbiquitin K48-linked |
| 6647-55 | SL017290 | P0CG48 | PolyUbiquitin K63 | PolyUbiquitin K63-linked |
| 6649-51 | SL012395 | O95631 | NET1 | Netrin-1 |
| 6653-58 | SL004838 | Q08722 | CD47 | Leukocyte surface antigen CD47 |
| 7624-19 | SL014896 | Q01484 | ANK2 | Ankyrin-2 |
| 7625-27 | SL008071 | P27348 | 14-3-3 protein theta | 14-3-3 protein theta |
| 7628-40 | SL012774 | Q96HD1 | CREL1 | Cysteine-rich with EGF-like domain protein 1 |
| 7638-30 | SL005403 | Q12907 | Lectin, mannose-binding 2 | Vesicular integral-membrane protein VIP36 |
| 7648-9 | SL017328 | Q00872 | MYPC1 | Myosin-binding protein C, slow-type |
| 7655-11 | SL002785 | P16860 | N-terminal pro-BNP | N-terminal pro-BNP |
| 8446-4 | SL011508 | P18509 | PACAP-27 | Pituitary adenylate cyclase-activating polypeptide 27 |
| 8447-11 | SL004269 | Q9UBU3 | ghrelin | Appetite-regulating hormone |
| 8450-36 | SL011498 | P18509 | PACAP-38 | Pituitary adenylate cyclase-activating polypeptide 38 |
| 8458-111 | SL005266 | P37840 | a-Synuclein | Alpha-synuclein |
| 8459-10 | SL003993 | P22004 | BMP-6 | Bone morphogenetic protein 6 |
| 8462-18 | SL000442 | P01241 | HGH | Somatotropin |
| 8464-31 | SL012517 | Q2I0M5 | RSPO4 | R-spondin-4 |
| 8465-52 | SL000346 | P09668 | Cathepsin H | Cathepsin H |
| 8467-9 | SL004837 | P08476 P09529 | Activin AB | Inhibin beta A chain:Inhibin beta B chain heterodimer |
| 8468-19 | SL000062 | P07288 | PSA | Prostate-specific antigen |
| 8469-41 | SL000466 | P18065 | IGFBP-2 | Insulin-like growth factor-binding protein 2 |
| 8470-213 | SL017528 | O60930 | RNase H1 | Ribonuclease H1 |
| 8476-11 | SL002762 | P10645 | CgA | Chromogranin-A |
| 8479-4 | SL000645 | P09238 | MMP-10 | Stromelysin-2 |
| 8480-29 | SL006527 | Q12805 | FBLN3 | EGF-containing fibulin-like extracellular matrix protein 1 |
| 8484-24 | SL000498 | P41159 | Leptin | Leptin |
| 8485-7 | SL017529 | Q14145 | KEAP1 | Kelch-like ECH-associated protein 1 |
| 9168-31 | SL003170 | Q9Y258 | Eotaxin-3 | C-C motif chemokine 26 |
| 9170-24 | SL001713 | Q16552 | IL-17 | Interleukin-17A |
| 9171-11 | SL016969 | P50461 | CSRP3 | Cysteine and glycine-rich protein 3 |
| 9172-69 | SL000526 | P22894 | MMP-8 | Neutrophil collagenase |
| 9173-21 | SL008094 | P36871 | PGM1 | Phosphoglucomutase-1 |
| 9175-48 | SL018891 | O60469 | DSCAM | Down syndrome cell adhesion molecule |
| 9176-3 | SL000001 | P15941 | MUC1 | Mucin-1 |
| 9177-6 | SL007306 | P58499 | FAM3B | Protein FAM3B |
| 9178-30 | SL004297 | Q02297 | NEUREGULIN-1 | Neuregulin-1 |
| 9180-6 | SL007217 | P38484 | INGR2 | Interferon gamma receptor 2 |
| 9182-3 | SL004733 | Q07954 | sLRP1 | Low-density lipoprotein receptor-related protein 1, soluble |
| 9183-7 | SL004475 | P17181 | IFN-a/b R1 | Interferon alpha/beta receptor 1 |
| 9185-15 | SL008591 | P04155 | TFF1 | Trefoil factor 1 |
| 9187-2 | SL013165 | P05114 | HMGN1 | Non-histone chromosomal protein HMG-14 |
| 9188-119 | SL003188 | Q07325 | MIG | C-X-C motif chemokine 9 |
| 9190-7 | SL008837 | P08962 | CD63 | CD63 antigen |
| 9191-8 | SL002602 | Q03403 | Trefoil factor 2 | Trefoil factor 2 |
| 9196-8 | SL005166 | P47929 | Galectin-7 | Galectin-7 |
| 9197-4 | SL008486 | O00182 | LEG9 | Galectin-9 |
| 9199-6 | SL018946 | P60604 | UB2G2 | Ubiquitin-conjugating enzyme E2 G2 |
| 9201-13 | SL004811 | P37802 | Transgelin-2 | Transgelin-2 |
| 9202-309 | SL008113 | P48047 | ATPO | ATP synthase subunit O, mitochondrial |
| 9204-33 | SL009210 | P01189 | Corticotropin-lipotropin | Pro-opiomelanocortin |
| 9207-60 | SL018887 | O95825 | QORL1 | Quinone oxidoreductase-like protein 1 |
| 9211-19 | SL003066 | P36955 | PEDF | Pigment epithelium-derived factor |
| 9212-22 | SL008381 | Q9UBX1 | CATF | Cathepsin F |
| 9213-24 | SL018900 | O95954 | FTCD | Formimidoyltransferase-cyclodeaminase |
| 9215-117 | SL018947 | Q9UHP3 | UBP25 | Ubiquitin carboxyl-terminal hydrolase 25 |
| 9216-100 | SL009948 | O15031 | PLXB2 | Plexin-B2 |

**Table S3** Gait speed and grip strength decline in CHS and FOS

|  | Gait speed, m/s | | Grip strength, kg | |
| --- | --- | --- | --- | --- |
|  | N | Mean ± SD | N | Mean ± SD |
| **CHS** |  |  |  |  |
| Baseline | 2854 | 0.927 ± 0.220 | 2854 | 27.972 ± 9.825 |
| 3-y changes | 2353 | -0.030 ± 0.225 | 2337 | -1.122 ± 4.980 |
| 6-y changes | 1888 | -0.086 ± 0.243 | 1894 | -2.842 ± 5.286 |
| **FOS** |  |  |  |  |
| Baseline | 868 | 1.235 ± 0.278 | 798 | 32.818 ± 13.002 |
| 7-y changes | 762 | -0.053 ± 0.312 | 703 | -2.257 ± 6.544 |

Abbreviation: CHS, Cardiovascular Health Study; FOS, Framingham Offspring Study

**Table S4** Significant SOMAscan proteins associated with gait speed decline by meta-analysis (fully adjusted model)

| SeqId | SomaId | UniProt | Target | Target full name | Estimates | Std Error | p-value |
| --- | --- | --- | --- | --- | --- | --- | --- |
| 4374-45 | SL003869 | Q99988 | GDF-15 | Growth/differentiation factor 15 | -0.0055 | 0.0007 | 7.61E-16 |
| 3045-72 | SL002704 | P21246 | PTN | Pleiotrophin | -0.0041 | 0.0007 | 7.31E-10 |
| 5315-22 | SL000052 | P45379 | Troponin T | Troponin T, cardiac muscle | -0.0039 | 0.0007 | 4.26E-08 |
| 2211-9 | SL000591 | P01033 | TIMP-1 | Metalloproteinase inhibitor 1 | -0.0035 | 0.0006 | 5.33E-08 |
| 5089-11 | SL005189 | P16871 | IL-7 Ra | Interleukin-7 receptor subunit alpha | 0.0034 | 0.0006 | 1.59E-07 |
| 6649-51 | SL012395 | O95631 | NET1 | Netrin-1 | -0.0033 | 0.0006 | 1.74E-07 |
| 3044-3 | SL003323 | P55774 | PARC | C-C motif chemokine 18 | -0.0029 | 0.0006 | 2.52E-06 |
| 2944-66 | SL005156 | P41271 | DAN | Neuroblastoma suppressor of tumorigenicity 1 | -0.0033 | 0.0007 | 3.37E-06 |
| 3799-11 | SL004867 | P07451 | Carbonic anhydrase III | Carbonic anhydrase 3 | -0.0028 | 0.0006 | 1.08E-05 |
| 2602-2 | SL001996 | O15123 | Angiopoietin-2 | Angiopoietin-2 | -0.0027 | 0.0006 | 1.66E-05 |
| 3438-10 | SL009324 | O95633 | FSTL3 | Follistatin-related protein 3 | -0.0027 | 0.0007 | 3.36E-05 |
| 3234-23 | SL010390 | Q76M96 | URB | Coiled-coil domain-containing protein 80 | -0.0027 | 0.0006 | 3.67E-05 |
| 11514-196 | SL004557 | P13987 | CD59 | CD59 glycoprotein | -0.0028 | 0.0007 | 5.02E-05 |
| 13094-75 | SL018509 | Q9BXY4 | RSPO3 | R-spondin-3 | -0.0026 | 0.0007 | 6.21E-05 |

Note: Estimates were from linear mixed effect models adjusted for age, sex, sex × time, race, clinic, education, height, weight, BMI, smoking status, eGFR, SBP, cognitive function, diabetes, CVD, cancer, COPD, arthritis, atrial fibrillation, and antihypertensive medicine. Significant threshold was 6.7×10^-5^.

**Table S5** Significant SOMAscan proteins associated with grip strength decline by meta-analysis (fully adjusted model)

| SeqId | SomaId | UniProt | Target | Target full name | Estimates | S.E. | | p-value |
| --- | --- | --- | --- | --- | --- | --- | --- | --- |
| 3799-11 | SL004867 | P07451 | Carbonic anhydrase III | Carbonic anhydrase 3 | -0.0755 | 0.0140 | 7.62E-08 | |
| 4541-49 | SL014092 | Q4KMG0 | CDON | Cell adhesion molecule-related/down-regulated by oncogenes | 0.0705 | 0.0135 | 1.61E-07 | |
| 13118-5 | SL011888 | Q9H4F8 | SMOC1 | SPARC-related modular calcium-binding protein 1 | -0.0750 | 0.0151 | 7.12E-07 | |
| 3397-7 | SL010616 | Q06124 | SHP-2 | Tyrosine-protein phosphatase non-receptor type 11 | -0.0645 | 0.0133 | 1.35E-06 | |
| 4374-45 | SL003869 | Q99988 | GDF-15 | Growth/differentiation factor 15 | -0.0691 | 0.0148 | 2.80E-06 | |
| 4272-46 | SL000539 | P06744 | PHI | Glucose-6-phosphate isomerase | -0.0577 | 0.0132 | 1.25E-05 | |
| 5480-49 | SL000563 | P13501 | RANTES | C-C motif chemokine 5 | 0.0585 | 0.0134 | 1.36E-05 | |
| 5508-62 | SL000344 | P07339 | Cathepsin D | Cathepsin D | -0.0603 | 0.0139 | 1.37E-05 | |
| 11514-196 | SL004557 | P13987 | CD59 | CD59 glycoprotein | -0.0637 | 0.0148 | 1.61E-05 | |
| 2201-17 | SL000403 | P39060 | Endostatin | Endostatin | -0.0596 | 0.0140 | 1.99E-05 | |
| 2612-5 | SL004759 | P55010 | eIF-5 | Eukaryotic translation initiation factor 5 | -0.0563 | 0.0133 | 2.23E-05 | |
| 2677-1 | SL002644 | P00533 | ERBB1 | Epidermal growth factor receptor | 0.0570 | 0.0137 | 3.29E-05 | |
| 4976-57 | SL013240 | P46108 | CRK | Adapter molecule crk | -0.0554 | 0.0134 | 3.68E-05 | |
| 4911-49 | SL003643 | P09211 | Glutathione S-transferase Pi | Glutathione S-transferase P | -0.0554 | 0.0134 | 3.76E-05 | |
| 5810-25 | SL005155 | P13385 | Cripto | Teratocarcinoma-derived growth factor 1 | -0.0547 | 0.0135 | 5.33E-05 | |
| 3285-23 | SL000310 | P00736 | C1r | Complement C1r subcomponent | 0.0540 | 0.0134 | 5.37E-05 | |
| 4474-19 | SL004070 | P62979 | Ubiquitin | Ubiquitin | -0.0536 | 0.0134 | 6.05E-05 | |
| 2789-26 | SL000525 | P09237 | MMP-7 | Matrilysin | -0.0544 | 0.0136 | 6.08E-05 | |
| 13093-6 | SL005430 | Q8WVN6 | SECTM1 | Secreted and transmembrane protein 1 | -0.0573 | 0.0144 | 6.48E-05 | |

Note: Estimates were from linear mixed effect models adjusted for age, sex, sex × time, race, clinic, education, height, weight, BMI, smoking status, eGFR, SBP, cognitive function, diabetes, CVD, cancer, COPD, arthritis, atrial fibrillation, and antihypertensive medicine. Significant threshold was 6.7×10^-5^.

**Table S6** Significant SOMAscan proteins associated with gait speed decline by meta-analysis (exclude prevalent stroke)

| SeqId | SomaId | UniProt | Target | Target full name | Estimates | Std Error | p-value |
| --- | --- | --- | --- | --- | --- | --- | --- |
| 4374-45 | SL003869 | Q99988 | GDF-15 | Growth/differentiation factor 15 | -0.0053 | 0.0007 | 1.82E-14 |
| 3045-72 | SL002704 | P21246 | PTN | Pleiotrophin | -0.0041 | 0.0007 | 1.01E-09 |
| 2211-9 | SL000591 | P01033 | TIMP-1 | Metalloproteinase inhibitor 1 | -0.0035 | 0.0007 | 1.06E-07 |
| 6649-51 | SL012395 | O95631 | NET1 | Netrin-1 | -0.0034 | 0.0006 | 1.13E-07 |
| 5315-22 | SL000052 | P45379 | Troponin T | Troponin T, cardiac muscle | -0.0037 | 0.0007 | 1.78E-07 |
| 5089-11 | SL005189 | P16871 | IL-7 Ra | Interleukin-7 receptor subunit alpha | 0.0034 | 0.0007 | 2.54E-07 |
| 3044-3 | SL003323 | P55774 | PARC | C-C motif chemokine 18 | -0.0029 | 0.0006 | 5.20E-06 |
| 2944-66 | SL005156 | P41271 | DAN | Neuroblastoma suppressor of tumorigenicity 1 | -0.0032 | 0.0007 | 7.36E-06 |
| 2602-2 | SL001996 | O15123 | Angiopoietin-2 | Angiopoietin-2 | -0.0027 | 0.0006 | 1.90E-05 |
| 3234-23 | SL010390 | Q76M96 | URB | Coiled-coil domain-containing protein 80 | -0.0028 | 0.0007 | 2.30E-05 |
| 3799-11 | SL004867 | P07451 | Carbonic anhydrase III | Carbonic anhydrase 3 | -0.0026 | 0.0007 | 5.51E-05 |
| 13094-75 | SL018509 | Q9BXY4 | RSPO3 | R-spondin-3 | -0.0027 | 0.0007 | 5.52E-05 |

Note: Estimates were from linear mixed effect models adjusted for age, sex, sex × time, race, clinic, education, height, weight, BMI, smoking status and eGFR. Significant threshold was 6.7×10^-5^.

**Table S7** Significant SOMAscan proteins associated with grip strength decline by meta-analysis (exclude prevalent stroke)

| SeqId | SomaId | UniProt | Target | Target full name | Estimates | S.E. | | p-value |
| --- | --- | --- | --- | --- | --- | --- | --- | --- |
| 4541-49 | SL014092 | Q4KMG0 | CDON | Cell adhesion molecule-related/down-regulated by oncogenes | 0.0701 | 0.0136 | 2.36E-07 | |
| 3799-11 | SL004867 | P07451 | Carbonic anhydrase III | Carbonic anhydrase 3 | -0.0722 | 0.0141 | 3.13E-07 | |
| 13118-5 | SL011888 | Q9H4F8 | SMOC1 | SPARC-related modular calcium-binding protein 1 | -0.0718 | 0.0151 | 2.01E-06 | |
| 3397-7 | SL010616 | Q06124 | SHP-2 | Tyrosine-protein phosphatase non-receptor type 11 | -0.0635 | 0.0135 | 2.40E-06 | |
| 4374-45 | SL003869 | Q99988 | GDF-15 | Growth/differentiation factor 15 | -0.0662 | 0.0148 | 8.23E-06 | |
| 2201-17 | SL000403 | P39060 | Endostatin | Endostatin | -0.0617 | 0.0140 | 1.02E-05 | |
| 5480-49 | SL000563 | P13501 | RANTES | C-C motif chemokine 5 | 0.0594 | 0.0136 | 1.29E-05 | |
| 5508-62 | SL000344 | P07339 | Cathepsin D | Cathepsin D | -0.0601 | 0.0141 | 1.91E-05 | |
| 11514-196 | SL004557 | P13987 | CD59 | CD59 glycoprotein | -0.0627 | 0.0148 | 2.25E-05 | |
| 4272-46 | SL000539 | P06744 | PHI | Glucose-6-phosphate isomerase | -0.0563 | 0.0133 | 2.40E-05 | |
| 2612-5 | SL004759 | P55010 | eIF-5 | Eukaryotic translation initiation factor 5 | -0.0565 | 0.0134 | 2.50E-05 | |
| 2789-26 | SL000525 | P09237 | MMP-7 | Matrilysin | -0.0565 | 0.0137 | 3.60E-05 | |
| 4474-19 | SL004070 | P62979 | Ubiquitin | Ubiquitin | -0.0554 | 0.0135 | 4.08E-05 | |
| 9188-119 | SL003188 | Q07325 | MIG | C-X-C motif chemokine 9 | -0.0555 | 0.0138 | 5.58E-05 | |
| 3485-28 | SL000283 | P61769 | b2-Microglobulin | Beta-2-microglobulin | -0.0729 | 0.0181 | 5.68E-05 | |

Note: Estimates are from linear mixed effect models adjusted for age, sex, sex × time, race, clinic, education, height, weight, BMI, smoking status and eGFR. Significant threshold was 6.7×10^-5^.

**Table S8** Significant SOMAscan proteins associated with gait speed decline by meta-analysis (adjusted for age × time interaction)

| SeqId | SomaId | UniProt | Target | Target full name | Estimates | S.E. | p-value |
| --- | --- | --- | --- | --- | --- | --- | --- |
| 5089-11 | SL005189 | P16871 | IL-7 Ra | Interleukin-7 receptor subunit alpha | 0.0037 | 0.0006 | 1.70E-08 |
| 4374-45 | SL003869 | Q99988 | GDF-15 | Growth/differentiation factor 15 | -0.0041 | 0.0007 | 2.68E-08 |
| 14150-7 | SL005177 | Q9UHA7 | IL-1F6 | Interleukin-36 alpha | -0.0029 | 0.0006 | 6.29E-06 |
| 2211-9 | SL000591 | P01033 | TIMP-1 | Metalloproteinase inhibitor 1 | -0.0027 | 0.0007 | 2.69E-05 |
| 5315-22 | SL000052 | P45379 | Troponin T | Troponin T, cardiac muscle | -0.0030 | 0.0007 | 2.76E-05 |

Note: Estimates are from linear mixed effect models adjusted for age, sex, age × time, sex × time, race, clinic, education, height, weight, BMI, smoking status and eGFR. Significant threshold was 6.7×10^-5^.

**Table S9** Significant SOMAscan proteins associated with grip strength decline by meta-analysis (adjusted for age × time interaction)

| SeqId | SomaId | UniProt | Target | Target full name | Estimates | Std Error | p-value |
| --- | --- | --- | --- | --- | --- | --- | --- |
| 3799-11 | SL004867 | P07451 | Carbonic anhydrase III | Carbonic anhydrase 3 | -0.0640 | 0.0142 | 6.35E-06 |
| 5480-49 | SL000563 | P13501 | RANTES | C-C motif chemokine 5 | 0.0599 | 0.0134 | 8.18E-06 |
| 3397-7 | SL010616 | Q06124 | SHP-2 | Tyrosine-protein phosphatase non-receptor type 11 | -0.0581 | 0.0134 | 1.43E-05 |
| 5508-62 | SL000344 | P07339 | Cathepsin D | Cathepsin D | -0.0579 | 0.0139 | 3.01E-05 |
| 4541-49 | SL014092 | Q4KMG0 | CDON | Cell adhesion molecule-related/down-regulated by oncogenes | 0.0566 | 0.0137 | 3.68E-05 |
| 2612-5 | SL004759 | P55010 | eIF-5 | Eukaryotic translation initiation factor 5 | -0.0542 | 0.0133 | 4.42E-05 |
| 4272-46 | SL000539 | P06744 | PHI | Glucose-6-phosphate isomerase | -0.0538 | 0.0132 | 4.59E-05 |

Note: Estimates were from linear mixed effect models adjusted for age, sex, age × time, sex × time, race, clinic, education, height, weight, BMI, smoking status and eGFR. Significant threshold was 6.7×10^-5^.

**Table S10** Top pathways associated with gait speed and grip strength decline using Reactome database.

| Pathway name | Entities | | | | Reactions | |
| --- | --- | --- | --- | --- | --- | --- |
|  | found | ratio | p-value | FDR* | found | ratio |
| **Gait speed** |  |  |  |  |  |  |
| Antagonism of Activin by Follistatin | 1/4 | 0.000345 | 0.004821 | 0.089845 | 1/2 | 0.000151 |
| Post-translational protein phosphorylation | 2/107 | 0.009233 | 0.007206 | 0.089845 | 1/1 | 0.000075 |
| RUNX1 regulates transcription of genes involved in WNT signaling | 1/6 | 0.000518 | 0.007224 | 0.089845 | 1/4 | 0.000301 |
| Regulation of Insulin-like Growth Factor (IGF) transport and uptake by Insulin-like Growth Factor Binding Proteins (IGFBPs) | 2/124 | 0.010700 | 0.009565 | 0.089845 | 1/14 | 0.001054 |
| Netrin mediated repulsion signals | 1/8 | 0.000690 | 0.009621 | 0.089845 | 4/4 | 0.000301 |
| **Grip strength** |  |  |  |  |  |  |
| Interleukin-3, Interleukin-5 and GM-CSF signaling | 3/48 | 0.004142 | 0.000055 | 0.015470 | 6/38 | 0.002860 |
| Signaling by EGFR | 3/52 | 0.004487 | 0.000070 | 0.015470 | 42/49 | 0.003688 |
| Signaling by Non-Receptor Tyrosine Kinases | 3/58 | 0.005005 | 0.000097 | 0.015470 | 11/53 | 0.003989 |
| Signaling by PTK6 | 3/58 | 0.005005 | 0.000097 | 0.015470 | 11/53 | 0.003989 |
| Collagen degradation | 3/64 | 0.005522 | 0.000129 | 0.015882 | 4/34 | 0.002559 |

Note: **Reactome** defines a 'reaction' as any event in biology that changes the state of a biological molecule. Binding, activation, translocation, degradation and classical biochemical events involving a catalyst are all reactions. * False Discovery Rate.
